# The human lymph node microenvironment unilaterally regulates T-cell activation and differentiation

**DOI:** 10.1371/journal.pbio.2005046

**Published:** 2018-09-04

**Authors:** Konstantin Knoblich, Sara Cruz Migoni, Susan M. Siew, Elizabeth Jinks, Baksho Kaul, Hannah C. Jeffery, Alfie T. Baker, Muath Suliman, Katerina Vrzalikova, Hisham Mehenna, Paul G. Murray, Francesca Barone, Ye H. Oo, Philip N. Newsome, Gideon Hirschfield, Deirdre Kelly, Steven P. Lee, Biju Parekkadan, Shannon J. Turley, Anne L. Fletcher

**Affiliations:** 1 Institute of Immunology and Immunotherapy, University of Birmingham, Edgbaston, Birmingham, United Kingdom; 2 Monash Biomedicine Discovery Institute and Department of Biochemistry and Molecular Biology, Monash University, Clayton, Australia; 3 Centre for Liver Research, Institute of Immunology and Immunotherapy, University of Birmingham, Edgbaston, Birmingham, United Kingdom; 4 Liver Unit, University Hospitals Birmingham National Health Service Foundation Trust, Edgbaston, Birmingham, United Kingdom; 5 Department of Gastroenterology and James Fairfax Institute of Paediatric Nutrition, The Children’s Hospital at Westmead, Sydney, Australia; 6 Institute of Cancer and Genomic Sciences, University of Birmingham, Edgbaston, Birmingham, United Kingdom; 7 Institute of Head and Neck Studies and Education, University of Birmingham, Edgbaston, Birmingham, United Kingdom; 8 Institute of Inflammation and Ageing, University of Birmingham, Edgbaston, Birmingham, United Kingdom; 9 National Institute for Health Research Liver Biomedical Research Unit at University Hospitals Birmingham National Health Service Foundation Trust and the University of Birmingham, Edgbaston, Birmingham, United Kingdom; 10 Department of Biomedical Engineering, Rutgers University, Piscataway, New Jersey, United States of America; 11 Department of Cancer Immunology, Genentech, South San Francisco, United States of America; National Cancer Institute, United States of America

## Abstract

The microenvironment of lymphoid organs can aid healthy immune function through provision of both structural and molecular support. In mice, fibroblastic reticular cells (FRCs) create an essential T-cell support structure within lymph nodes, while human FRCs are largely unstudied. Here, we show that FRCs create a regulatory checkpoint in human peripheral T-cell activation through 4 mechanisms simultaneously utilised. Human tonsil and lymph node–derived FRCs constrained the proliferation of both naïve and pre-activated T cells, skewing their differentiation away from a central memory T-cell phenotype. FRCs acted unilaterally without requiring T-cell feedback, imposing suppression via indoleamine-2,3-dioxygenase, adenosine 2A Receptor, prostaglandin E2, and transforming growth factor beta receptor (TGFβR). Each mechanistic pathway was druggable, and a cocktail of inhibitors, targeting all 4 mechanisms, entirely reversed the suppressive effect of FRCs. T cells were not permanently anergised by FRCs, and studies using chimeric antigen receptor (CAR) T cells showed that immunotherapeutic T cells retained effector functions in the presence of FRCs. Since mice were not suitable as a proof-of-concept model, we instead developed a novel human tissue–based in situ assay. Human T cells stimulated using standard methods within fresh tonsil slices did not proliferate except in the presence of inhibitors described above. Collectively, we define a 4-part molecular mechanism by which FRCs regulate the T-cell response to strongly activating events in secondary lymphoid organs while permitting activated and CAR T cells to utilise effector functions. Our results define 4 feasible strategies, used alone or in combinations, to boost primary T-cell responses to infection or cancer by pharmacologically targeting FRCs.

## Author summary

The lymph node microenvironment contains an abundance of immune cells that interact with and within an intricate structural framework created by fibroblastic reticular cells. In mice, fibroblastic reticular cells are known to regulate T-cell activation, proliferation, and function, but in humans, they are poorly understood. We investigated interactions between human T cells and human fibroblastic reticular cells from tonsils and lymph nodes. When T cells were activated in the presence of human fibroblastic reticular cells, their proliferation and differentiation were reduced, without altering effector T-cell function, shown through cytokine production. We identified 4 molecular mechanisms that were responsible, concurrently used by all human fibroblast donors tested, and reversible upon addition of specific inhibitors to the cocultures. To establish the relevance of this finding outside of in vitro coculture, we showed that T-cell proliferation was increased in live human tonsil tissue slices when the fibroblastic reticular cell inhibitors were added. This work demonstrates that human fibroblastic reticular cells regulate T-cell activation and provides new information on the mechanisms used, which may be useful to design clinical strategies that improve T-cell responses.

## Introduction

Stromal cells create specialised lymphoid support compartments within secondary lymphoid organs. The signals they feed leukocytes have profound effects in many aspects of activation, proliferation, and differentiation [1].

Fibroblastic reticular cells (FRCs) construct the internal segregated structure of secondary lymphoid organs by acting as a scaffold for lymphocyte migration and secreting chemokine C-C motif ligand 19 (CCL19) and chemokine C-C motif ligand 21 (CCL21) to bring T cells and dendritic cells to the central T-cell zone and chemokine C-X-C motif ligand 13 (CXCL13) to bring B cells to outer B cell zones. Lymphocyte survival is further supported through secretion of survival factors interleukin 7 (IL-7) and B cell activating factor (BAFF) [2,3].

Several papers demonstrated that mouse lymph node–derived FRCs reduce T-cell proliferation. In mice, when T cells have been activated less than 15 h, cyclooxygenase-2 (COX2)-driven prostaglandin E2 (PGE2) is suppressive [4], while comparative neutralisation experiments showed that nitric oxide plays a larger role past 15 h, when most T-cell division occurs [5–7]. T-cell function is impaired, shown through reduced interferon gamma (IFNγ) production [6–7]. Effects on memory T-cell differentiation have not been assessed in mice.

Human FRCs are still almost entirely unstudied, though it has been shown that podoplanin (PDPN^+^) cells analogous to mouse FRCs are found in human secondary lymphoid organs and that they secrete extracellular matrix components as well as CCL21 [8,9]. A recent study, citing as-yet unpublished data, said that human FRCs do not produce nitric oxide in response to IFNγ activation [9]. We therefore questioned whether prior mouse FRC research accurately modelled human FRC biology. The effects of FRCs on human T cells are unknown, and their mechanism/s of action have not been tested, though COX2 is expressed [4,9].

The role of human FRCs in T-cell regulation is likely to be highly relevant to human health. Mouse studies show far-reaching effects of FRCs for immunity against influenza and other pathogens [3,10,11], and it is hypothesised that suppression of effector T-cell activation within lymph nodes reduces immune-mediated pathology against the lymph node structure [1,12]. Accordingly, virally infected FRCs are associated with T-cell persistence and chronic viral infection [12].

Here, we show that human FRCs block proliferation and modulate differentiation of newly activated naïve human T cells, without requiring T-cell feedback. Suppression was constitutive, and we identified 4 molecular mechanisms operating simultaneously: indoleamine-2,3-dioxygenase (IDO), COX1 and 2 enzymes responsible for PGE2 production, adenosine 2A receptor (A2AR), and transforming growth factor beta (TGFβ). Coinhibition of these factors reversed FRC-mediated suppression in vitro and permitted us to observe T-cell activation on a living tonsil slice.

It is important to first understand the key cells and molecules involved in regulating T-cell activation during inflammation, infection, cancer, and autoimmunity in order to treat immune-mediated pathologies and immune deficiencies. Here, we show that human FRCs, via 4 pathways—which are druggable individually or in small combinations—are a likely pharmacological target to boost the primary immune response.

## Results

To test the effect of FRCs on naïve T-cell activation, we isolated and culture-expanded FRCs from either cadaver-origin lymph nodes or live-donor tonsils using a published digestion protocol [13] or through explant culture. Cultured FRCs used in this study were identified as CD45^−^, CD31^−^, PDPN^+^ cells across multiple passages, and they expressed genes and proteins characteristic of published FRC phenotypes from freshly isolated mouse and human FRCs [14,15] (S1 Fig), including expression of PDPN, CXCL12, α smooth muscle actin (αSMA; ACTA2), lymphotoxin beta receptor (LTBR), platelet-derived growth factor receptor (PDGFR) α and β, vimentin (VIM), detectable but low levels of BAFF, mucosal vascular addressin cell adhesion molecule 1 (MADCAM1), Desmin, CD34, and receptor activator of NF-κB ligand (RANKL). As expected, chemokines CCL21 and CCL19 were switched off by cultured cells; transcription in mice has been shown to be regulated by lymphatic flow [16]. We identified a distinct subset of stromal cells present in freshly isolated tonsil but missing from our cultures; these were CD45^−^CD31^−^EpCAM- CD90^+^ CD73^−^ PDPN^−^ cells (S1 Fig) with unknown function.

**Fig 1:**
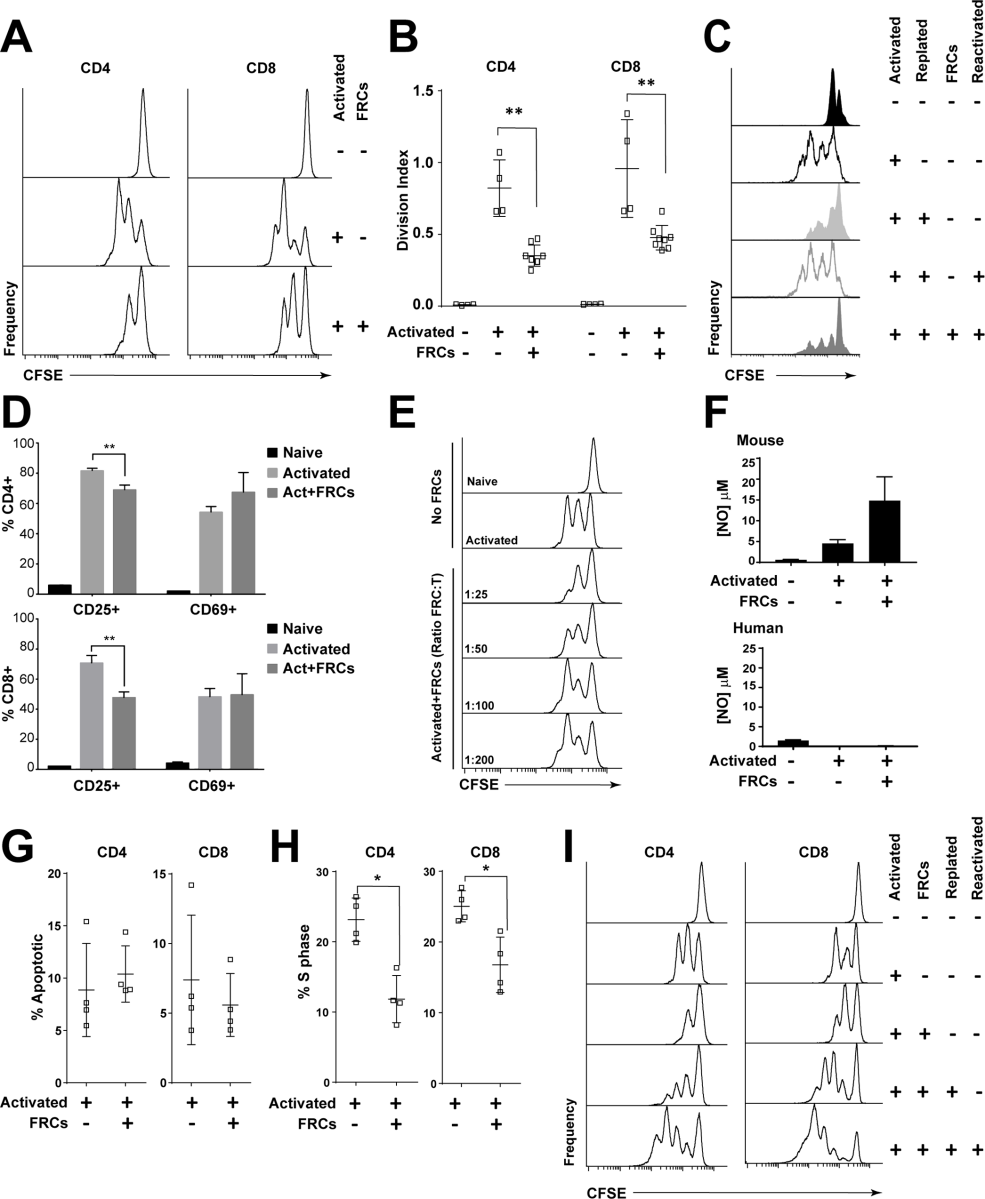
Human FRCs inhibit T-cell proliferation via secretion of soluble factors. A. CFSE-labelled PBMCs (5 × 10^5^) were cultured with or without 2 × 10^4^ FRCs in 96-well plates, with or without anti-CD3/CD28/CD2-coated activation beads. After 96 h, cells were harvested and analysed using flow cytometry. Histograms depict T-cell CFSE staining on plots gated for CD3 and CD4 or CD8. Data are representative of at least 6 FRC and PBMC donors and 10 independent experiments. B. Division index of gated CFSE+ T cells, which were cultured for 96 h unstimulated or stimulated with anti-CD3/CD28/CD2-coated beads, with or without FRCs. Data depict 2 PBMC donors and 4 FRC donors from 2 independent experiments. ** *P* < 0.01; 2-tailed Mann-Whitney test; error bars depict SD. C. CFSE-labelled PBMCs (5 × 10^5^) were cultured with or without anti-CD3/CD28/CD2-coated activation beads for 48 h or 96 h. At 48 h, some wells were harvested, washed, and replated with or without anti-CD3/CD28/CD2-coated activation beads (reactivation) and with or without FRCs. All cells were harvested at 96 h for flow cytometric analysis. Plots gated on CD3, CD4, or CD8 and CFSE. Data are representative of 3 FRC donors and 3 PBMC donors from 3 independent experiments. D. PBMCs (5 × 10^5^) were stimulated using anti-CD3/CD28/CD2-coated activation beads with or without precultured FRCs for 96 h and analysed for activation markers CD25 and CD69. Error bars represent SD. Data depict a single experiment, which is representative of *N* = 8 FRC donors and *N* = 3 PBMC donors from 3 independent experiments. ** *P* < 0.01; unpaired 2-tailed *t* test; error bars depict SD. E. CFSE-labelled PBMCs (5 × 10^5^) were cultured with or without anti-CD3/CD28/CD2-coated activation beads and with or without varying concentrations of FRCs as indicated for 96 h. All cells were harvested at 96 h for flow cytometric analysis. Data are representative of 2 FRC donors, 3 PBMC donors, and 3 independent experiments. Plots gated on CD3+ cells. F. The activation assay described in A. was performed and supernatant harvested at 72 h (mouse) or 96 h (human). Nitrite, as a breakdown product of nitric oxide release, was measured using the Griess assay. Figure represents cumulative result of 2 independent experiments. Error bars represent SD. G-I. CFSE-labelled PBMCs (5 × 10^5^) were cultured with or without 2 × 10^4^ FRCs in 96-well plates, with or without anti-CD3/CD28/CD2-coated activation beads. G. At 96 h, cell cycle analysis of CD4 and CD8 T cells was performed using BrdU and 7AAD. The percentage of cells expressing a phenotype sub-G1 was defined as apoptotic. Data depict 1 FRC donor and 1 PBMC donor per experiment, from 4 independent experiments. Mean and SD are shown. H. At 96 h, cell cycle analysis of CD4 and CD8 T cells using BrdU and 7AAD to assess percentage of cells in S phase. Data depict 4 FRC donors and 1 PBMC donor from 1 experiment and are representative of 4 independent experiments. * *P* < 0.05; 2-tailed Mann-Whitney test. Mean and SD are shown. I. After 48 h, some wells of activated T cells cultured in the presence of FRCs were harvested, washed, and replated with or without stimulant (anti-CD3/CD28/CD2-coated activation beads). At 96 h, all wells were harvested and analysed using flow cytometry. Plots gated for CD3, CD4, or CD8 and CFSE. Figure is representative of 2 FRC donors with 1 PBMC donor per experiment, from 2 independent experiments. Data used in the generation of this figure can be found in S1 Data. 7AAD, 7-aminoactinomycin D; BrdU, bromodeoxyuridine; CFSE, carboxyfluorescein succinimidyl ester; FRC, fibroblastic reticular cell; PBMC, peripheral blood mononuclear cell; S phase, DNA-synthesis phase.

We activated carboxyfluorescein succinimidyl ester (CFSE)-labelled T-cells from peripheral blood mononuclear cells (PBMCs) in the presence of human FRCs and found that they underwent fewer divisions after 96 h (Fig 1A and 1B). We then activated CFSE-labelled PBMCs and allowed T-cells to divide freely for 48 h. Cells were harvested and plated with or without FRCs and reactivated for a further 48 h. While reactivated T cells proliferated freely, the addition of FRCs after the initial activation and prior to the second activating stimulus imposed a significant brake on proliferation (Fig 1C). Thus, FRCs could hamper the proliferation of both naïve and pre-activated, actively divided T cells.

Next, we examined surface marker expression changes in responding T cells. After 96 h of activation with or without FRCs, T cells showed normal up-regulation of early activation marker CD69, but significantly fewer T cells up-regulated the interleukin 2 receptor alpha (IL-2Ra) chain, CD25 (Fig 1D). Together with data showing that FRCs could halt the proliferation of pre-activated T cells, these results suggested that the suppressive mechanism occurred well downstream of T-cell receptor ligation and did not involve steric hindrance. Suppression was responsive to dose (Fig 1E), and human FRCs did not produce nitric oxide when stimulated (Fig 1F).

We questioned whether FRCs may be inducing apoptosis or permanently anergising responding T cells. Cell cycle analysis showed no increase in apoptosis in either CD4^+^ or CD8^+^ T cells (Fig 1G). Instead, in the presence of FRCs, fewer T cells entered the DNA-synthesis phase (S phase), compared to T cells that were not cocultured with FRCs (Fig 1H), with no change to G0/G1 or G2/M phase (S2 Fig). Separation of activated, suppressed T cells from FRCs, followed by reactivation alone in culture, showed that T cells were not permanently anergised by activation in the presence of FRCs (Fig 1I).

We probed the nature of the suppression mechanism by screening small molecular inhibitors, agonists, and blocking/neutralising antibodies in a similar coculture assay, in which CFSE-labelled T cells were activated in the presence or absence of FRCs and/or inhibitors and analysed after 4 days.

**Fig 2:**
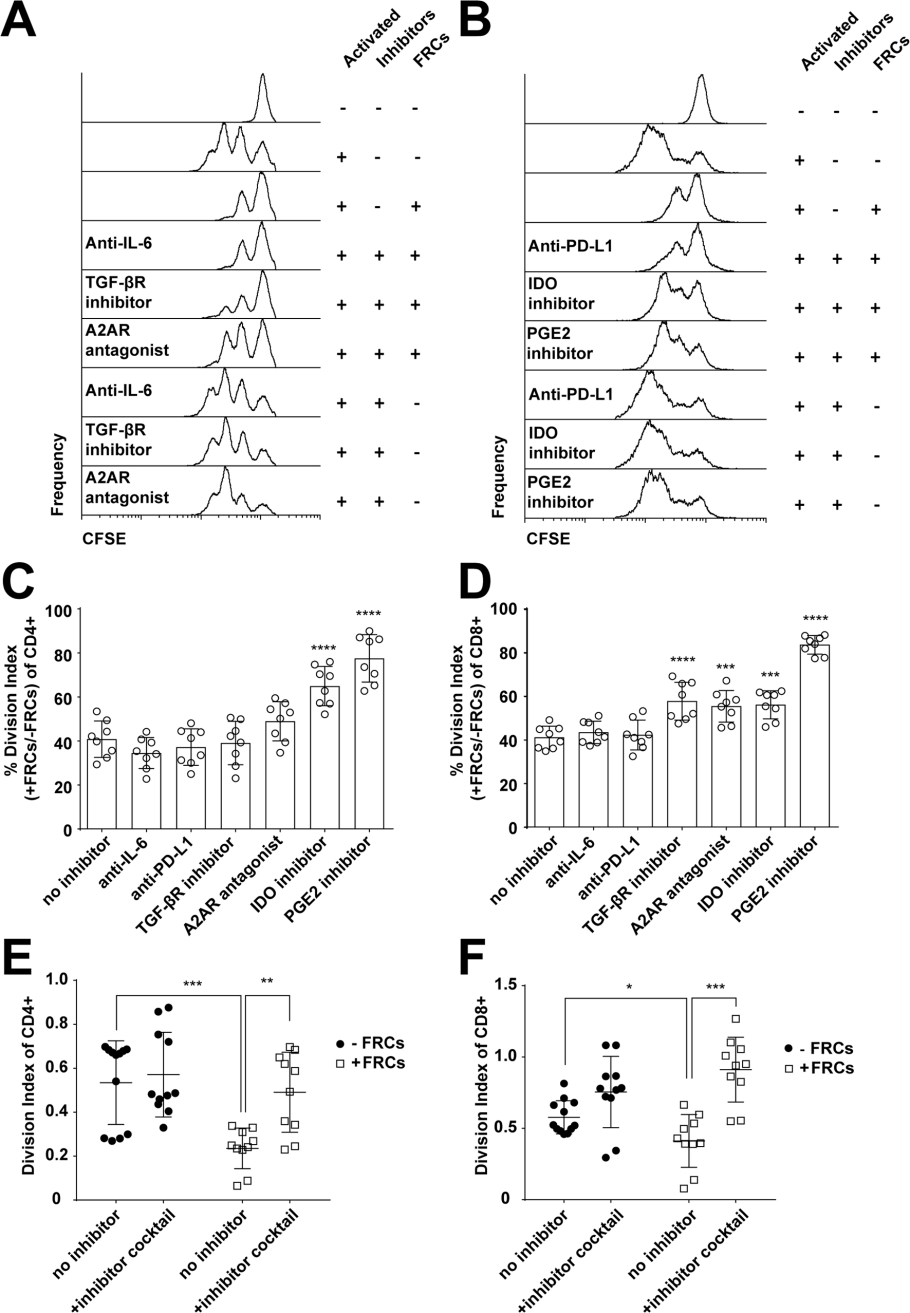
FRCs mediate their effects through COX1/2, IDO, TGFβR, and A2AR. A., B. CFSE-labelled PBMCs (5 × 10^5^) were stimulated with PHA-L/IL-2 with or without precultured FRCs and in the presence of inhibitors for IL-6, TGFβR, or A2AR (A) or PD-L1, IDO, or PGE2 (B). Some wells were left unstimulated. After 96 h, cells were harvested and analysed by flow cytometry. Plots gated as CD3+. Figure depicts 4 FRC donors and 2 PBMC donors from 2 independent experiments and is also reflective of results obtained using anti-CD3/CD28/CD2-coated activation beads. C, D. Data from A, B were analysed for normalised division index of CD4+ (C) or CD8+ (D) T cells; 100% = maximal division of stimulated T cells in the absence of FRCs; 0% = minimal division, from unstimulated T cells. All groups shown were stimulated in the presence of FRCs, without inhibitor (no inhibitor) or with inhibitors for IL-6, PD-L1, TGFβR, A2AR, IDO, or PGE2. Data depict 4 FRC donors and 2 PBMC donors from 2 independent experiments. Error bars represent SD. **** *P* > 0.0001, *** *P* > 0.001. Statistics derived from 1-way ANOVA with Dunnett’s multiple comparison test. E, F. CFSE-labelled PBMCs were stimulated with anti-CD3/CD28/CD2-coated activation beads, with or without precultured FRCs, and in the presence or absence of inhibitor cocktail (A2AR agonist, TGF-βR inhibitor, IDO inhibitor, COX1/2 inhibitor). Division index of CD4 (E) and CD8 (F) T cells is shown. Figure depicts 3 independent experiments each with 2–4 FRC donors and 1 PBMC donor per experiment. Mean and SD are shown. *** *P* < 0.001, ** *P* < 0.01, * *P* < 0.05. Statistics derived from a 2-tailed unpaired *t* test. Data used in the generation of this figure can be found in S1 Data. A2AR, adenosine 2A receptor; COX1/2, cyclooxygenase-1/2; FRC, fibroblastic reticular cell; IDO, indoleamine-2,3-dioxygenase; IL-6, interleukin 6; PBMC, peripheral blood mononuclear cell; PD-L1, programmed cell death ligand 1; PGE2, prostaglandin E2; PHA-L/IL-2, phytohaemagglutinin-L/interleukin 2; TGFβRI, transforming growth factor beta receptor type 1.

Inhibition or blockade of TGFβ receptor, IDO, COX1/2, and A2AR signalling all restored T-cell proliferation (Fig 2A–2D) to varying degrees, while interleukin 6 (IL-6) and programmed cell death ligand 1 (PD-L1) inhibition had no effect (Fig 2A–2D), and inhibitors did not alone significantly alter T-cell differentiation phenotypes (S3 Fig).

All 4 mechanisms were utilised in all donors but to varying degrees, with no conserved predominance (S4 Fig). This imposed challenges in how to most appropriately assess the overall effect of FRC suppression on T-cell biology in a meaningful manner, given human variance and the potential for redundancy. Donor-to-donor variance was minimal when all 4 mechanisms were targeted at once, and since all mechanisms were operational in all donors, we reasoned that this was the best means of assessing the net biological impact of FRCs on T cells. We therefore chose to suppress all mechanisms simultaneously to investigate downstream effects on T cells.

To assess whether these 4 molecular targets were together sufficient to entirely block FRC suppression, we created an inhibitory cocktail of all 4 inhibitors and treated PBMCs that were exposed to stimulatory signals for 96 h. In the presence of FRCs, proliferation of both CD4 and CD8 T cells occurred at control levels (Fig 2E and 2F). The inhibitor cocktail did not alone significantly increase T-cell proliferation in the absence of FRCs (Fig 2E and 2F).

**Fig 3:**
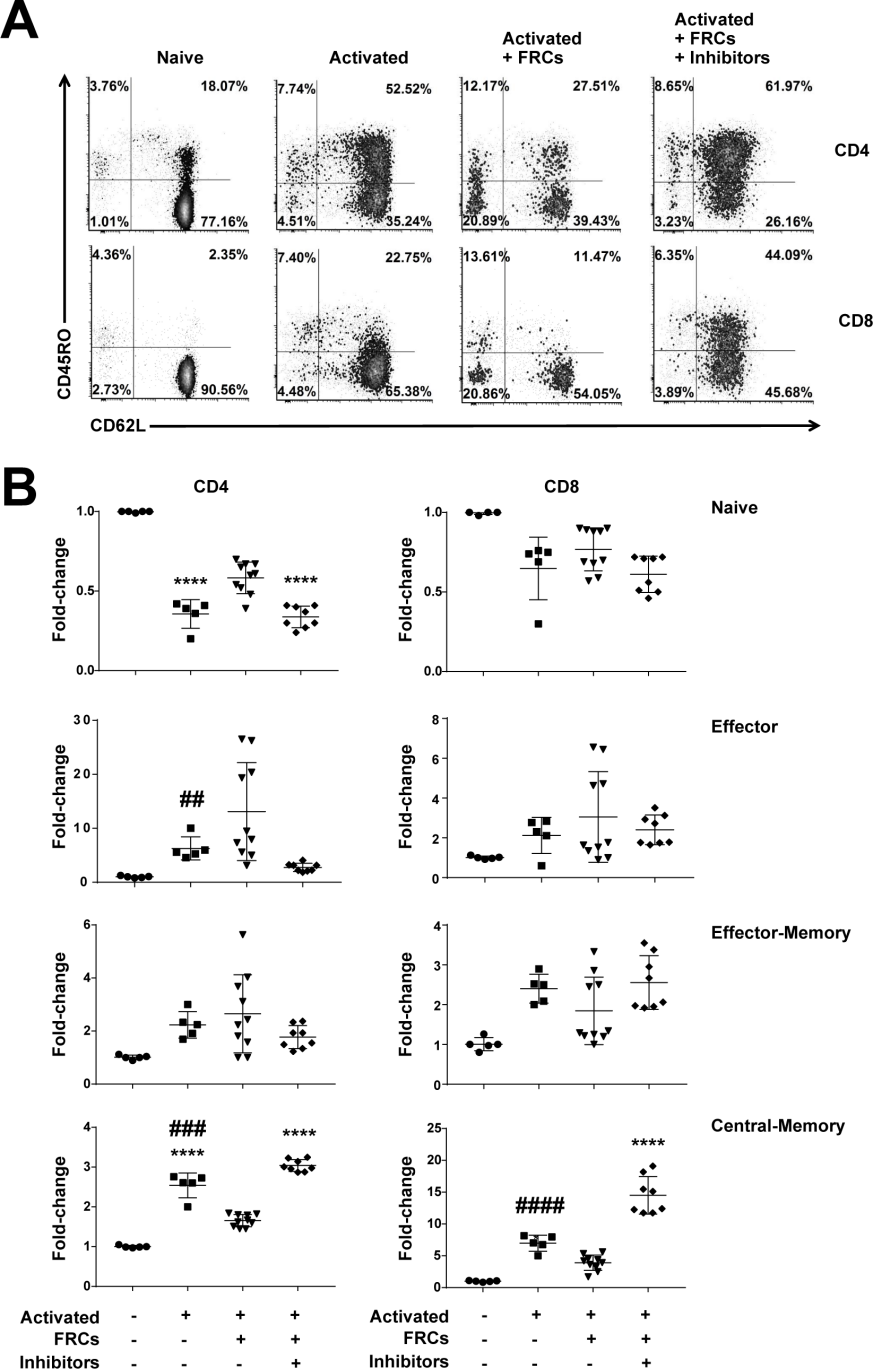
FRCs constrain differentiation of T cells with a central memory phenotype. A. CFSE-labelled PBMCs (5 × 10^5^) were stimulated with anti-CD3/CD28/CD2-coated beads, with or without precultured FRCs, and in the presence or absence of the inhibitor cocktail. Some wells were left unstimulated. After 96 h, cells were harvested and analysed by flow cytometry. Plots gated for CD3, CD4, or CD8; CD62L; and CD45RO. Figure is representative of 3 FRC donors and 3 PBMC donors from a total of 3 independent experiments. B. Fold-change analysis of FRC effect on T-cell differentiation from experiments conducted as described in A. A star (*) defines significance compared to untreated; a hash (#) defines significance compared to activated+FRCs+inhibitors. Two symbols refers to *P* < 0.01; 3 symbols to *P* < 0.001; 4 symbols to *P* < 0.0001, using a 1-way ANOVA with Sidak’s multiple comparisons test. Data depict 3 independent experiments, each with 2–4 FRC donors and 1 PBMC donor. Data used in the generation of this figure can be found in S1 Data. FRC, fibroblastic reticular cell; PBMC, peripheral blood mononuclear cell.

Strikingly, the presence of FRCs influenced the fate of differentiating naive CD4^+^ and CD8^+^ T-cell populations (CD62L+CD45RO−). Their presence during T-cell activation decreased differentiation of CD4+ and CD8+ T cells to a central memory phenotype (CD62L+CD45RO+) while increasing the proportion of CD4+ naïve (CD62L+CD45RO−) T cells. Effector (CD62L−CD45RO−) and effector memory phenotype T cells (CD62L− CD45RO+) and CD8+ naïve T cells were not affected (Fig 3A and 3B). Memory phenotype cells were further profiled by expression of CD27 and by relative proliferation; both subsets yielded expected phenotypes (S5 Fig). Blockade of TGFβR, IDO, COX1/2, and A2AR signalling reversed the effects (Fig 3A and 3B). Results were comparable regardless of whether anti-CD2/3/28-coated beads or PHA-L/IL-2 was used as the activating stimulus.

**Fig 4:**
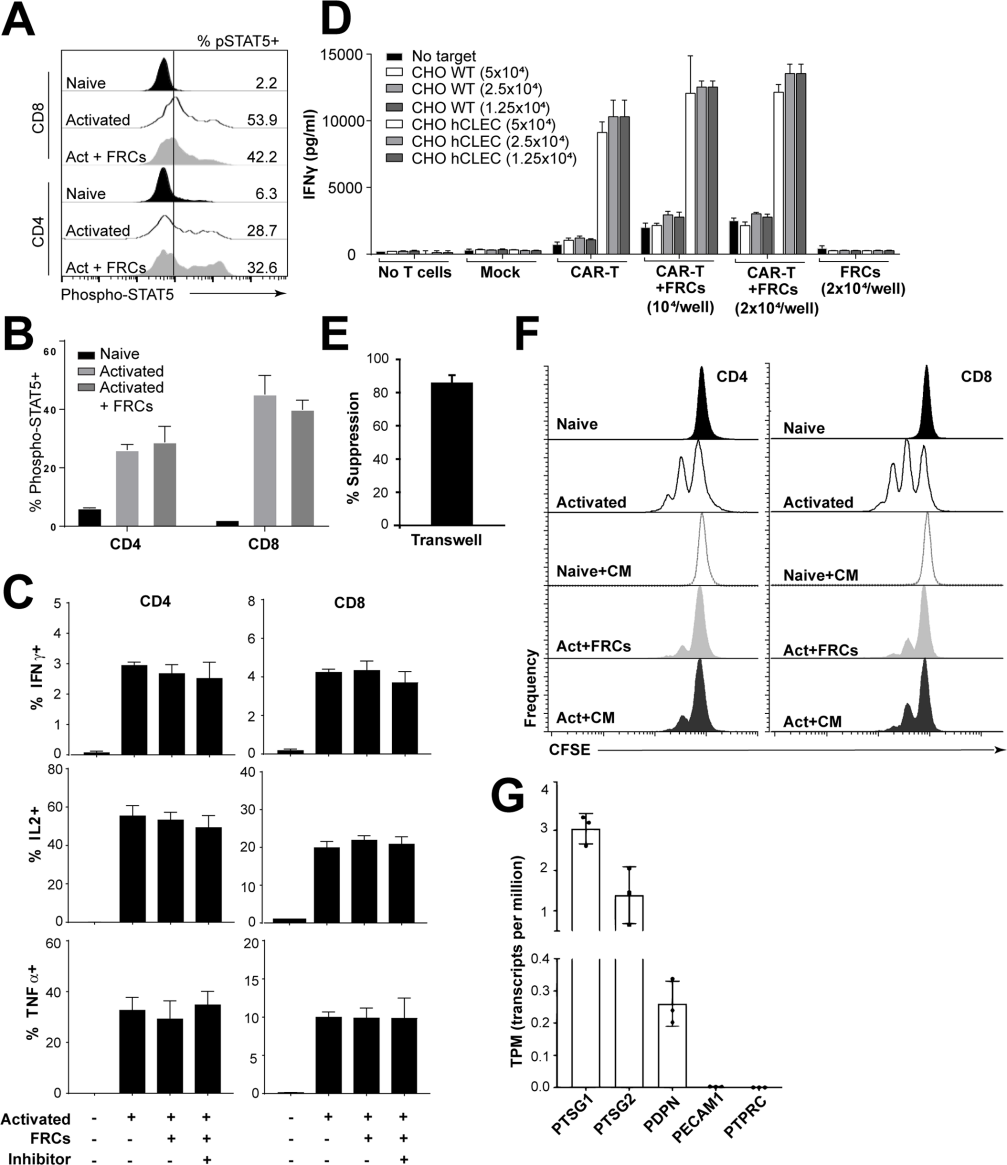
FRCs act unilaterally to suppress T cells. A, B. PBMCs (5 × 10^5^) were stimulated with anti-CD3/CD28/CD2-coated beads with or without precultured FRCs and in the presence or absence of the inhibitor cocktail for 48 h. Cells were then washed to remove stimulant and allowed to rest for 24 h before anti-CD3/CD28/CD2-coated bead reactivation for 15 min. Cells were then harvested and stained for pSTAT5 using the PhosFlow kit according to the manufacturer’s instructions. A depicts histograms gated on CD3+ T cells. B depicts aggregate data from 1 experiment of *N* = 1 PBMC donor and *N* = 2 FRC donors; error bars depict SD. Data are representative of *N* = 4 FRC donors and *N* = 2 PBMC donors from 2 independent experiments. C. PBMCs (5 × 10^5^) were stimulated by anti-CD3/CD28/CD2-coated beads with or without precultured FRCs and in the presence or absence of the inhibitor cocktail for 96 h. Brefeldin A was added for the last 4 h of activation. Cells were harvested; stained for IFNγ, IL-2, or TNFα; and analysed by flow cytometry. Bar graphs depict *N* = 3 FRC donors and *N* = 1 PBMC donor. Data represent 2 independent experiments. Mean and SD depicted. D. Mock-transduced or transduced CART cells were stimulated with target CHO cells expressing antigen (hCLEC) or irrelevant protein (WT), in the presence or absence of FRCs at 2 dilutions. FRCs alone or no T-cell groups were negative controls. Eighteen h after coculture, IFNγ concentration was tested using ELISA. Data represent *N* = 2 independent experiments. E. FRCs were cultured in 24-well plates for 24 h prior to introduction of CFSE-labelled T cells in a transwell. After 48 h of activation with anti-CD3/CD28/CD2-coated beads, T cells were harvested, and percent suppression was assessed by flow cytometry by taking the ratio of percent T cells divided in the presence of a 1 μm transwell, divided by the percent divided in the absence of the transwell × 100. Mean and SD depicted from *N* = 2 FRC donors and 2 PBMC donors from 2 independent experiments. F. CFSE-labelled PBMCs were stimulated with anti-CD3/CD28/CD2-coated beads, with or without precultured FRCs. Some wells were left unstimulated, and some stimulated wells had a 1:2 dilution of FRC CM added. After 96 h, cells were harvested and analysed by flow cytometry. Plots gated on CD3, CD4, or CD8 and CFSE. Data are representative of *N* = 4 FRC donors and *N* = 2 PBMC donors from 2 independent experiments. CM was derived from *N* = 2 FRC donors, each used in 2 independent experiments. G. RNA-Seq data from cultured (passage 3) human tonsil–derived FRCs. *N* = 3 unrelated FRC donors. *Y* axis represents normalised expression of genes in transcripts per million. Data used in the generation of this figure can be found in S1 Data. CAR, chimeric antigen receptor; CHO, Chinese hamster ovary; CM, conditioned medium; FRC, fibroblastic reticular cell; hCLEC, human C-type lectin domain family 14 member A; IFNγ, interferon gamma; IL-2, interleukin 2; PDPN, podoplanin; PECAM1, platelet and endothelial cell adhesion molecule 1; PBMC, peripheral blood mononuclear cell; pSTAT5, phosphorylated signal transducer and activator of transcription 5; PTPRC, protein tyrosine phosphatase receptor type C; PTSG1, prostaglandin-endoperoxide synthase 1; PTSG2, prostaglandin-endoperoxide synthase 2; RNA-seq, RNA sequencing; TNFα, tumour necrosis factor alpha; WT, wild type.

Since CD25 is the alpha chain of the IL-2R, and since we observed selective inhibition of CD25 expression by FRCs (Fig 1D), which was present as early as 24 h after stimulation and did not occur in the presence of inhibitors (S6 Fig), this led us to question whether FRC-mediated effects on IL-2 signalling were evident. Coculture with FRCs did not alter signal transducer and activator of transcription 5 (STAT5) phosphorylation (Fig 4A and 4B), leading us to conclude that this signalling pathway was not mechanistically important; we tested other signalling molecules phosphorylated extracellular signal–regulated kinase 1/2 (pERK1/2), phosphorylated STAT1 (pSTAT1), phosphorylated STAT3 (pSTAT3), phosphorylated STAT6 (pSTAT6), and p38 mitogen-activated protein kinase (MAPK) and found no mechanistic insight within T cells (S7 Fig). IL-2 production, as a downstream transcriptional target of IL-2 signalling, was also not altered (Fig 4C). Together, these results demonstrated that an inhibition of the IL-2 signalling pathway was not driving T-cell suppression. Accordingly, the proportion of T regulatory cells (Tregs) was also neither increased nor decreased in stimulated cultures (S8A and S8B Fig), and depletion of CD25+ cells did not prevent FRC-mediated suppression (S8C Fig), suggesting that the effect of FRCs did not occur via cross-talk with Tregs.

Production of IFNγ and tumour necrosis factor alpha (TNFα) were also unaffected by coculture with FRCs (Fig 4C), suggesting that while FRCs inhibit activation and differentiation of T cells, once T cells are active, their effector functions are not impaired. This finding was confirmed using antigen-activated chimeric antigen receptor (CAR) T cells (Fig 4D) and is a finding not reproduced in mouse models, in which IFNγ production is reduced following FRC coculture [6].

Given the broad differences here observed between mice and humans, we decided to test whether T-cell cross-talk with human FRCs was required for or important to suppression. In mice, having T cells and FRCs in close proximity is important, since separation by transwell blocks the majority of the suppressive effect of FRCs [5,6]. Residual suppression in the presence of the transwell is likely to be due to secretion of PGE2 [4]. Unlike mouse FRCs, suppression was retained when FRCs were separated from T cells by a cell-impermeable transwell barrier (Fig 4E). Moreover, FRC-conditioned media diluted 1:2 in complete media suppressed T-cell activation indistinguishably from FRCs (Fig 4F), showing that FRCs secrete suppressive factors constitutively and that, unlike mouse FRCs [5,6] or human mesenchymal stromal cells [7], they do not require cross-talk from activated T cells. Accordingly, steady-state cultured FRCs expressed high levels of PGE2 synthesis enzymes COX1 (*PTSG1*) and COX2 (*PTSG2*) (Fig 4G). COX1 is usually a constitutive source of PGE2, while COX2 is more commonly inflammation inducible yet was expressed in otherwise unstimulated FRC cultures (Fig 4G) [4]. COX2, A2AR, and transforming growth factor beta receptor type 2 (TGFβR2) protein staining was detectable on ERTR7+ T-zone FRCs (TRCs) present in frozen tonsil tissue sections (S9 Fig) from patients who were not suffering from infection at the time of surgery. We were unable to detect IDO staining, suggesting that this protein alone may be inducible under acute inflammatory conditions.

As a method of cross-confirmation to show that FRCs preproduce suppressive factors prior to seeing T cells, we preincubated FRCs with inhibitors, prior to a wash step, and then added T cells and an activation stimulus. T cells stimulated with pre-inhibited FRCs had high CD25 expression after 24 h, similar to FRCs with inhibitors and higher than uninhibited FRCs (S6 Fig), as also shown in Fig 1D. CD25 expression was used as a biomarker for FRC suppression of T cells, as T-cell division was not measurable until 48 h, and the pre-incubation step was, because of receptor turnover, not effective beyond 24 h.

Next, we looked for a means to validate these in vitro results. Activation of T cells in situ on secondary lymphoid organ tissue slices has not previously been shown, to our knowledge. We decided to test whether this could be due to a suppressive effect of stromal cells, by stimulating T cells in situ within slices of freshly donated tonsil, in the presence or absence of the inhibitory cocktail. Tonsils were obtained within 2–4 h of surgery from healthy donors, immediately sectioned, and then incubated in media containing phytohaemagglutinin-L (PHA-L) and recombinant human interleukin 2 (rhIL-2), with or without the inhibitor cocktail added. After 96 h, T cells were imaged or isolated by mechanical disruption and analysed by flow cytometry.

**Fig 5:**
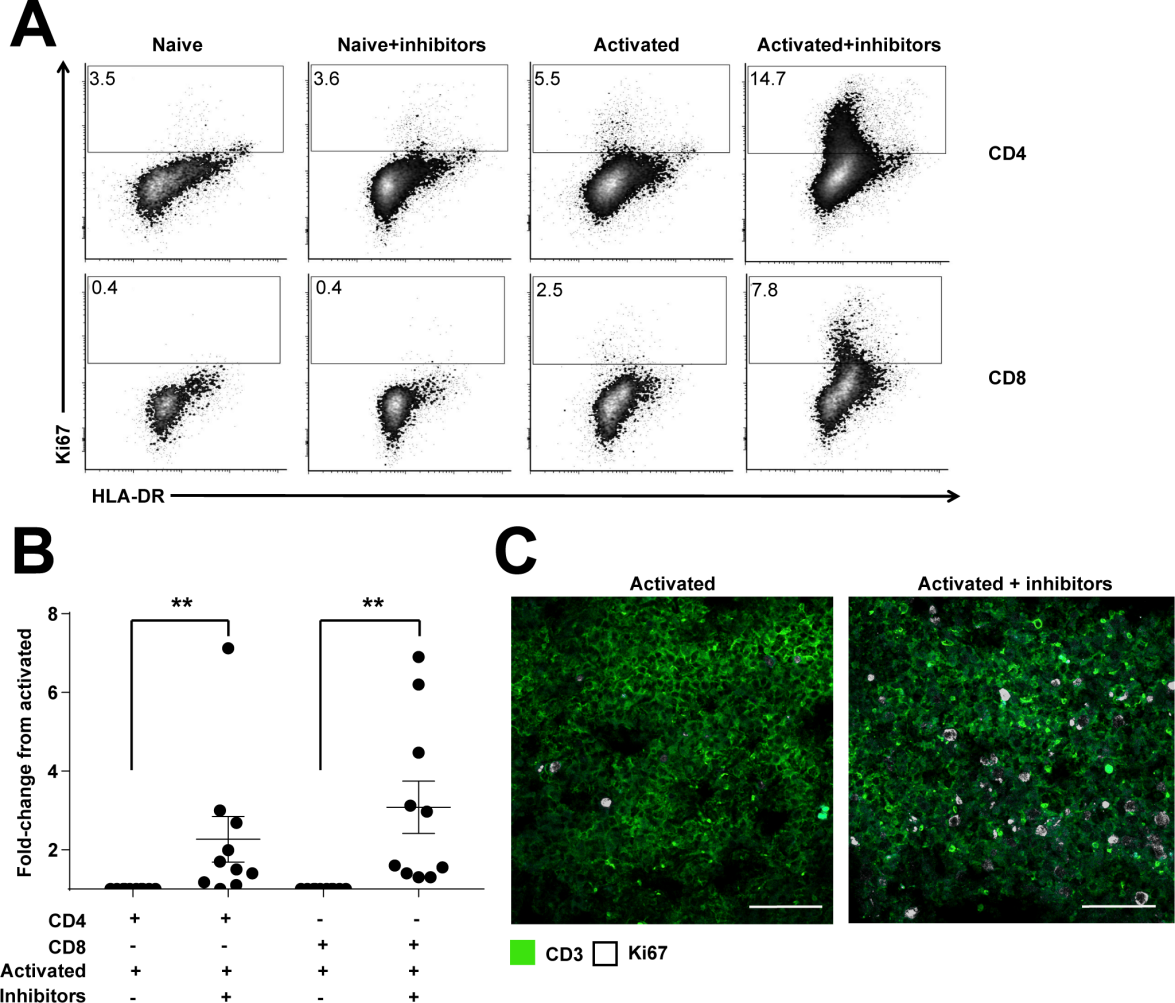
*Ex vivo* activation of T cells. Fresh tonsil tissue slices were incubated with or without stimulant (PHA-L/IL-2) and either with or without the presence of the inhibitor cocktail. All slices were incubated for 96 h. A. Slices were harvested and mechanically disrupted before staining for flow cytometry. Plots were gated for CD3, CD4, or CD8 and Ki67, with HLA-DR as a counterstain. Percentage of CD3+CD4+ or CD3+CD8+ T cells that are Ki67+ is shown. Figure depicts 1 tonsil donor, representative of *N* = 10 tonsil donors from at least 5 independent experiments. B. Fold-change analysis of T-cell activation (Ki67) from tissue slices analysed via flow cytometry. *Y* axis represents fold change from stimulated controls. A 2-tailed Wilcoxon signed rank test was used to obtain *P*-values comparing activated+inhibitor to activated; ** *P* < 0.01. Error bars represent SEM. Figure depicts *N* = 10 tonsil donors from at least 5 independent experiments. C. Confocal microscopy of tonsil tissue slices stimulated with PHA-L + IL-2 for 90 h with or without inhibitor cocktail. CD3 in green, Ki67 in white. Scale bar in white represents 50 μm. Representative example of 2 independent experiments. Data used in the generation of this figure can be found in S1 Data. PHA-L/IL-2, phytohaemagglutinin-L/interleukin 2.

A significantly higher proportion of CD4+ and CD8+ T cells were observed in active cell cycle (Ki67+) in the presence of the inhibitory cocktail, shown through flow cytometry (Fig 5A and 5B) and immunofluorescence (Fig 5C). Controls showed baseline expression (Fig 5A–5C). Immunofluorescent imaging showed increased Ki67 staining in the T-cell zone when inhibitors and the activating stimulus were both present (Fig 5C). Ki67 staining robustly reproduced in vitro results and demonstrated that it is possible to inhibit stromal-induced T-cell suppression in situ.

Taken together, these results show that human FRCs strongly influence the activation and differentiation of naïve T cells by constraining initial proliferation and skewing their differentiation away from a central memory phenotype without altering effector cytokine production or signalling. Mechanisms of action involved PGE2, COX1/2, TGFβ, and A2AR. Coinhibition of these factors permitted us to observe T-cell activation on a living tonsil slice for the first time, to our knowledge.

## Discussion

Identifying the cells and molecules that regulate T-cell activation during inflammation, infection, cancer, and autoimmunity is a fundamental first step towards creating effective therapies for immune-mediated pathologies and immune deficiencies. Human immunological studies are commonly carried out in vitro, with validation in mice. Here, we show that the presence of a human microenvironment influences T-cell activation both in vitro and in situ, with important functional and mechanistic differences from previous observations in mice.

Previous studies using mouse lymph nodes have established that FRCs are important for regulation of T-cell proliferation, largely through provision of nitric oxide [5–7] with an early role for PGE2 [4,6]. Changes to differentiation were not observed in mice, but FRCs did compromise effector cytokine production and therefore T-cell function [6].

By contrast, our results showed key differences to mice. Human FRCs utilised 4 pathways independent of nitric oxide to control T-cell proliferation and differentiation. Inhibiting TGFβR, A2AR, IDO, and COX1/2 completely reversed the suppressive effect of FRCs and restored differentiation of T cells with a central memory phenotype to normal levels. Similarly, the inhibition of these targets in situ using tonsil slices allowed T cells to overcome the prohibitive effect that stromal cells imposed.

Unlike mice, bidirectional T-cell signalling to FRCs was not required for T-cell suppression or for expression of A2AR, TGFBR, and COX2 protein by TRCs. Expression of IDO was not detected, and it is expected that IDO is induced by acute inflammation, as reported in dermal and bone marrow fibroblasts [17]. It will be of interest in the future to explore the factors and kinetics governing its induction.

Once activated, polyclonal and CAR T cells both showed normal effector cytokine secretion, which again differs from mice. It is currently unclear whether these are bona fide biological differences between mice and humans or due to the preferential study of immunologically naïve mice raised in a specific pathogen-free environment. It would be interesting to see whether FRCs from mice that have undergone several rounds of a self-limiting infection would constitute a more representative model for human FRCs.

The finding that T-cell function in the presence of FRCs is maintained is highly relevant to cancer immunotherapy. Secondary lymphoid organs are a primary tumour site for lymphoma and leukaemia and a prominent metastatic site for many other cancers. They are an important site for transfused CAR T-cell activity, with one study showing CAR T cells heavily infiltrating lymph nodes of patients with lymphoma at >30% of T cells [18] and another showing CAR T transcripts detectable in lymph nodes up to 3 mo post-infusion and at higher levels in lymph nodes than in blood [19]. Mouse data suggested that FRCs would limit IFNγ production by effector T cells; these results show that human FRCs only impose effects on naïve T-cell proliferation and differentiation and not effector function. Secretion of key effector cytokines IFNγ, TNFα, and IL-2 is unchanged in the presence of FRCs after T cells are activated, which is the case for CAR T therapy.

Much like nitric oxide as a mechanism in mouse FRCs, human FRC molecular mechanisms are extremely complex, and the precise effects on T cells are not easily elucidated, despite their clear biological importance and decades of intense study. TGFβR signalling, the COX1 enzyme, A2AR, and IDO all have well-described suppressive effects on T-cell activation. But apart from IDO, none are uniformly anti-inflammatory; rather, each factor is capable of shaping the T-cell response in a complex manner dependent on the stimulus, costimulating factors, and the microenvironmental cytokine milieu [20,21]. As such, inhibitors targeting the molecules profiled in this study have been investigated for highly diverse applications. A2AR inhibitors, for example, are used to treat rheumatoid arthritis (methotrexate [22]) while being investigated as an immunomodulatory treatment for cancer, for which the goal is inhibition of immunosuppression [23]. The level of TGFβ signalling to naïve T cells is an important factor in enforcing their quiescence, and naïve T cells in patients with autoimmunity have reduced expression of TGFβRI and increased capacity for T-cell proliferation [24], but TGFβ blockade enhances vaccine and immunotherapy responses [25]. With similar complexity, murine FRCs have a role in deletional and suppressive tolerance [5,6,26] while promoting healthy immune responses [3,10].

IDO has a well-defined role in T-cell suppression. It oxidises tryptophan to kynurenine metabolites [27], which both deprives effector T cells of tryptophan, inducing proliferative arrest [28], and exposes them to immunosuppressive kynurenine, which can impair T-cell growth and survival [29]. IDO is a well-described mechanism of tumour immune evasion in mice [28] and shows direct effects in human T cells [30–32], though a phase 3 trial of combination IDO inhibitor and programmed cell death protein 1 (PD-1) inhibition recently failed to improve progression-free survival compared to PD-1 inhibitor immunotherapy alone (clinicaltrials.gov identifier: NCT02752074). IDO affects the earliest stages of TCR signalling through down-regulation of Vav1 and inhibition of F-actin reorganisation [30,31], as well as inhibition of the mammalian target of rapamycin (mTOR) pathway [29], and, as we observed, prevents cells progressing to S phase of the cell cycle [33].

COX1 and COX2 are enzymes involved in prostaglandin synthesis. Our inhibitor is capable of blocking both, but our data show that COX1 is a major mediator of FRC suppression, since FRC-conditioned media strongly suppressed T cells, and cultured FRCs only expressed COX1 constitutively. COX1 can also be involved in the earliest inflammatory events, after which COX2 becomes the predominant inflammatory isoform [34]. In humans, PGE2 is the most abundant member of the prostanoid family, and most PGE2 is secreted by professional antigen-presenting cells (APCs) and stromal cells [21]. PGE2 is capable of mediating diverse effects depending on stimuli that are not well understood, but its role in suppression of T-cell activation and proliferation has been reported since 1971 [35], with newer studies also describing skewed differentiation [36] and induction of a suppressive phenotype in non-Treg CD4+ T cells, which were capable of suppressing the proliferation of other T cells undergoing activation [37]. Reported mechanisms include down-regulation of CD25, up-regulation of CD46, and altered responses to costimulation [36].

Extracellular purinergic mediators, such as adenosine triphosphate (ATP) and adenosine, are powerful immunomodulators. They signal through A2AR as an important step in the resolution of inflammation, providing well-described suppressive influences on the function of T cells, dendritic cells, macrophages, mast cells, platelets, natural killer (NK) cells, B cells, fibroblasts, and neutrophils to prevent excessive tissue pathology [20,38,39]. Accordingly, methotrexate is a clinically important A2AR antagonist used to treat rheumatoid arthritis and other autoimmune diseases [22]. Adenosine production is increased in inflammation and in low-oxygen-tension microenvironments, and activation of A2AR increases intracellular cAMP, which inhibits cytokine responses. Accordingly, inhibition of A2AR awakens tumour-reactive CD8+ T cells in mouse models [40]. The oxygen tension in human lymphoid organs is likely to be low: murine lymphoid organs exhibit low oxygen tension in vivo at 0.5%–4.5% oxygen, which impacts upon the speed at which effector T cells differentiate [41], and while data on healthy lymph nodes are lacking, low partial pressures are reported for other human organs and tissues [42].

Certain human T-cell subsets are known to express CD39, which converts ATP to AMP [43], while human FRCs express high levels of CD73, which converts AMP to adenosine. A2AR signals reduce secretion of proinflammatory IL-1β and IL-6 [22], both of which are produced at high levels by FRCs in response to inflammation [11], and increases production of collagen I [44].

TGFβ is a highly evolutionarily conserved immunomodulatory molecule [45]. Our inhibitor blocked signalling receptor component activin receptor-like kinase 5 (ALK5; TGFβRI), whose signals are transduced by phosphorylation of SMAD2 and SMAD3 proteins [46]. TGFβRI^−/−^ mice develop a lethal inflammatory disease [47], and neutralising TGFβ increases antitumour responses of CD8^+^ T cells [48]. However, TGFβ can impose both pro- and anti-inflammatory functions in responding human T cells, depending on their differentiation state and inflammatory cytokines encountered [45]. As an anti-inflammatory agent, TGFβ inhibits CD4+ and CD8+ T-cell clonal expansion and differentiation and inhibits the activation of high-affinity T cells [49–51], relevant to our findings, and it also promotes the survival of lower-affinity T cells. These effects can occur in a paracrine fashion by acting on APCs and other cells and by reducing CD25 expression [51]. TGFβ signalling blocks the clonal expansion of T cells in vivo and blocks differentiation of T helper 1 (Th1), T helper 2 (Th2), and cytotoxic lymphocyte (CTL) T cells in favour of T helper 17 (Th17; in the presence of IL-6) or peripheral Treg (pTreg; in the presence of retinoic acid and IL-2) [50].

The described pathways could potentially also link together. PGE2 can increase the expression of IDO in dendritic cells [52]. Similarly, signalling pathways downstream of PGE2 ligands EP2–4 involve cAMP, which is derived from ATP [21], potentially linking the suppressive actions of COX1 inhibition and A2AR inhibition. However, these potential interactions are very poorly studied and understood and require extensive further study.

Suppression of T-cell proliferation within secondary lymphoid organs seems paradoxical, but these and other results [5–7] show it is conserved in mice and humans, despite utilising different mechanisms. Mouse studies clearly show that it operates in vivo, in isolated FRCs in response to inflammation [5]. Here, we show that it operates in vitro using human cells and in situ in human tissues and that FRCs also secrete key suppressive factors in the absence of current inflammation. One hypothesis is that this mechanism helps prevent bystander damage to stromal cells in a lymph node teeming with inflammatory cytokines and antigen [1,12], and the mechanisms described all have well-charted effects in the resolution of inflammation [39]. Accordingly, viral infection of FRCs is associated with viral persistence in mice [12], and a loose association has been observed in humans and rhesus macaques in studies of HIV and Ebola virus, though studies are correlative [1].

While human FRCs did not affect the secretion of effector cytokines from activated cells, they did impose effects on the proliferation of activated cells. FRCs added to pre-activated, rapidly dividing cultures halted their division, and when activated, suppressed T cells are removed from the suppressive influence of FRCs, they begin rapidly dividing within 24 h, despite being washed thoroughly to remove the inflammatory cytokine milieu, and without being given a new activating stimulus. Thus, the effects of FRCs are not limited to naïve T cells.

This raises the possibility of pharmacologically targeting FRCs as a means to promote a stronger immune response. It would be valuable to study whether FRC inhibition could benefit patient groups who do not mount a robust response to vaccination, or to boost responses to cancer vaccines.

The in situ activation assay revealed a clear disconnect between the study of human immunology in vitro, in which T cells are frequently activated in isolation, and in situ, in the presence of the microenvironment. Our work highlights the importance of considering the microenvironment for in vitro human immunology studies. Conditions that robustly activated T cells in culture were entirely insufficient to activate T cells in situ. Addition of the inhibitor cocktail significantly increased T cell activation in situ but not to in vitro levels, suggesting the presence of additional physical or chemical inhibitory factors that warrant further study. Similarly, in mice, rare FRCs were observed up-regulating the suppression-mediating nitric oxide synthase 2 (NOS2) enzyme during an in vivo T-cell immune response [5], which was sufficient to robustly impair T-cell activation. These processes are clearly observable yet subject to kinetics and fate decisions we have yet to fully understand—suppressive yet finely tuned to permit T-cell activation and foster immunity.

The in situ activation assay has potential to test drugs in development for their effects on T-cell activation and proliferation. A technical challenge, requiring further study, is the ability to isolate slices from equivalent areas of tissue. The proportion of naïve T cells within a single donor varied hugely from slice to slice. As such, it was not yet possible to use this method to assess differences in subtler immunophenotypic changes, such as differentiation status. Another important caveat is our lack of transcriptomic data from freshly isolated human FRCs. Cultured FRCs in this study lacked a PDPN-low/negative subset that was present in freshly isolated tonsil FRCs, and the function of this subset in humans is not yet known.

Together, this work suggests that FRCs utilise druggable targets (individually or in combinations) with the potential to boost the generation of new immune responses within secondary lymphoid organs—for example, following vaccination of relevant patient groups or for the treatment or prevention of malignancy.

## Materials and methods

### Ethics statement

All tissues were obtained from consenting donors from the National Disease Research Interchange (NDRI) resource centre or Human Biomaterials Resource Centre (HBRC), Birmingham (HTA licence 12358, 15/NW/0079), under project approval number REC_RG_HBRC_12–071. All tissues were obtained and utilised in accordance with institutional guidelines and according to the principles expressed in the Declaration of Helsinki.

### Human tissues

Human tonsils were obtained from presently healthy children and adults undergoing routine tonsillectomy for a medical history of recurrent infection or obstructive sleep apnoea. Human blood was obtained from healthy adult donors. Human lymph nodes were procured from cadaveric donors, transported intact in DMEM on ice, and processed for flow cytometry or cell culture within 24 h. All tissues were obtained and utilised in accordance with institutional guidelines.

### Single-cell primary cultures

Tonsils and lymph nodes were enzymatically digested using a published protocol [13] or grown through explant culture and used at passage 1–3. Briefly, tissues were cut into small pieces and grown in a low volume of complete media with antibiotics (alpha-MEM with 10% FBS, with penicillin, streptomycin, and a mycoplasma elimination reagent) for 24 h to allow adhesion to the tissue culture plate. Following this, tissues were covered with media containing antibiotics and grown for 5 days to permit fibroblasts to emerge. Tissue was then discarded and cells culture-expanded in complete media without antibiotics. Using this method, a monolayer of >99% pure FRCs was achieved within 2 wk. Ten-fold expansion was taken to equal 1 passage. FRCs were defined as CD45−, CD31−, PDPN+. Very rarely, cultures down-regulated expression of PDPN after passage 3; this did not affect their transcriptome or suppressive T-cell interactions (not shown); nonetheless, such cultures were not used experimentally to ensure uniformity.

### T-cell activation assay

FRCs (2 × 10^4^) were plated in a 96-well flat-bottom plate in complete media (alpha-MEM, 10% FBS) and allowed to adhere for 4 h. FRCs were always used in experiments prior to passage 3. Mononuclear leukocytes were isolated from whole blood using a density gradient and then counted using a haemocytometer and Trypan Blue viability dye. Where stated, T cells were purified using the Pan T-cell isolation kit (Miltenyi Biotec) according to the manufacturer’s instructions and at a purity >90%, or CD25 depleted (Miltenyi Biotec) according to the manufacturer’s instructions and at a purity >90%. Mononuclear leukocytes or T cells (5 × 10^5^) were added, together with a stimulant: either CD2/3/28 T-cell activation beads (2 beads/T cell) (Miltenyi Biotec) or PHA-L (1 μg/ml) + rhIL-2 (100 U), as stated in figure legends. Inhibitors were added at the following concentrations: SB431542 10 μM (TGFβ signalling pathway inhibitor through blockade of ALK5, 7, 4, Sigma), Indomethacin 5 μM (Cox1/2 and PGE2 synthesis inhibitor, Sigma), SCH 58261 10 μM (A2AR inhibitor, Sigma), 1-methyl-D-Tryptophan (1-MT) 1 mM (Indoleamine-2,3-dioxygenase inhibitor, Sigma). SCH 58261 and SB431542 10 μM were stored in DMSO. PGE2 inhibitor was reconstituted in ethanol; 1-MT was reconstituted in methanol and pH adjusted to 7.0. The final volume per well was 200 μl, and all cells and inhibitors were resuspended in complete media without antibiotics. All 4 inhibitors used together at stated concentrations are referred to as the ‘inhibitor cocktail’.

### CAR T-cell activation assay

FRCs (1–2 × 10^4^) and Chinese hamster ovary (CHO; 5 × 104–1.25 × 10^5^) cells were plated in a 96-well flat-bottom plate in complete media (DMEM, 10% FBS with IL-2 [25 IU/ml]) and allowed to adhere for 4 h. CRT-3 (1 × 10^5^) or mock-transduced T cells were added and incubated for 18 h at 37°C/5% CO_2_. Culture supernatants were collected at 18 h, and the levels of IFNγ were titrated in culture supernatants using the ELISA method. Briefly, plates (Nunc) were coated with anti-human IFNγ Ab diluted in coating buffer (0.75 μg/ml) and incubated at 4°C overnight. After blocking the wells using buffer containing PBS plus 0.05% (v/v) Tween 20 and 0.1% (w/v) bovine serum albumin (BSA), supernatants were added to each well. Biotin-labelled mAb in incubation buffer was added to each well, and streptavidin-HRP was used as enzyme. The reaction was developed using 3,3′,5,5′-tetramethylbenzidine (TMB) substrate and stopped by adding 1 M hydrochloric acid. The plates were washed after each step using PBS with 0.05% (v/v) Tween 20. Reading was performed using a microplate automatic reader (Biorad) at a wavelength of 450 nm.

### In situ T-cell activation

Tonsils <2 h from surgery were sliced into multiple 0.4–0.6 mm sections using a sterilised carbon-fibre microtome blade or embedded in low-melting-point agarose and sectioned using a vibratome, collected into ice-cold PBS. Sections were randomised between groups and cultured in complete media containing PHA-L (1 μg/ml) + rhIL-2 (100 U), with or without the inhibitor cocktail described above. Multiple slices were used per treatment group. After 96 h, each slice was pushed through a cell strainer to create a single-cell suspension and stained for flow cytometry or embedded in OCT buffer and snap-frozen for sectioning and imaging. To minimise differences arising from sectioning different areas of tissue, data from multiple slices were averaged to obtain a single data point per donor.

### Flow cytometry

Cells were harvested at stated time points and stained for 20 min in FACS buffer (PBS with 2% FCS and 2 mM EDTA) using antibodies as described in S1 Table. Cells were fixed and permeabilised using a commercial kit (BD) and then stained for intracellular proteins using the following antibodies: cells were resuspended for flow cytometry, filtered through 100 μm mesh, and acquired using flow cytometry. Analysis utilised commercial analysis software (TreeStar or DeNovo Software). tSNE analysis was performed using 1,000 iterations, with perplexity 30 and theta 0.5, displaying a proportional number of events.

### Imaging

Human tonsils were embedded in O.C.T. Compound (Sakura) and then flash frozen using dry ice. Then, 10–12 mm transverse sections were generated on a cryostat (Bright Instrument Company) and collected on adhesive slides (Leica). Sections were air-dried for 2 h at room temperature (RT) and then fixed in cold acetone for 25 min. Sections were air-dried overnight for immediate immunolabelling or stored at −80°C until further use. Frozen tonsil sections were air-dried for 15 min at RT and rehydrated for 5 min with 1X PBS. Sections were then permeabilised for 15 min with 0.3% Triton X-100 (ThermoFisher Scientific) and washed 3 times with 1X PBS. Sections were blocked for 1 h in 1% BSA (Sigma-Aldrich) and 5% goat serum in PBS in a humidified chamber. Sections were incubated overnight at 4°C with primary antibodies diluted in 1% BSA. After incubation, sections were washed 3 times with 1X PBS and then incubated with secondary antibody for 1 h. Secondary antibodies included goat anti-rat Alexa 546 (ThermoFisher), goat anti-rat Alexa 546 (ThermoFisher), goat anti-rat goat Alexa 647 (ThermoFisher), donkey anti-rabbit Alexa 488 (ThermoFisher), goat anti-rabbit Alexa 647 (ThermoFisher), donkey anti-mouse DyLight 594 (ThermoFisher), and donkey anti-goat Alexa 555 (ThermoFisher). Sections were then incubated with DAPI for 1 min, followed by 3 additional washes with 1X PBS. Negative controls utilised incubation with PBS with relevant serum or relevant isotype, followed by secondary antibody. Finally, sections were mounted in antifade mountant (ThermoFisher Scientific) for imaging. Immunofluorescence images were taken with a confocal microscope Zeiss LSM 880, using ZEN Pro imaging system.

### RNA-Seq

Tonsil-derived FRCs from 3 donors (in-house) and bone marrow–derived MSCs from 3 donors (Lonza and expanded in-house) were grown in culture to P3 in the presence of hFGF (4 μg/ml) and harvested in logarithmic growth phase. Total RNA was extracted using an RNA extraction kit (Qiagen) and purified using a cleanup kit (Qiagen). Samples were quality tested using an Agilent Bioanalyzer 2100 and the Agilent RNA 6000 nano kit. RIN numbers for all samples ranged from 9.5 to 10. Samples were then sent to BGI (Hong Kong) for library preparation and sequencing. Briefly, library preparation utilised poly-A enrichment followed by Ribozero depletion. Samples were run in a high-performance sequencing machine (Illumina) over 2 lanes, resulting in approximately 60 million reads per sample. Data were then trimmed for adapter sequences before analysis. Bioinformatics alignment and further analysis was done in-house using commercial software (Partek). For a visual representation of gene expression, TPM was used for normalisation. The heatmap was made using Morpheus (https://software.broadinstitute.org/morpheus). Data are accessible at monash.figshare.com doi: 10.4225/03/5a2dae0c9b455.

### Statistics

Data were tested for normality using D’Agostino and Pearson normality test. Normally distributed data of 2 groups were compared using an unpaired *t* test, or of 3 or more groups using an ANOVA with a multiple comparison test, as described in figure legends. When data were not normally distributed, 2 comparisons were made using a Mann-Whitney test. When fold-change data were compared to a normalised value of 1, a 2-tailed Wilcoxon signed rank test was used. *P* < 0.05 was taken as significant.

## Supporting information

S1 Data Raw numbers used to construct primary and supplemental figures. (XLSX)

S1 Table Primary antibodies list. A list of primary antibodies used in flow cytometry and imaging. Columns from left to right represent antigen antibody was raised for; conjugated fluorescent label of the antibody, if applicable; antibody isotype; antibody clone; source of the antibody; and application the antibody was used for.
(XLSX)

S1 Fig Comparison between freshly isolated and cultured FRCs. A. Human tonsil–derived FRCs, at various passages or freshly isolated, were gated as CD45^−^ CD31^−^ EpCAM^−^ and assessed by flow cytometry for expression of αSMA, CD73, CD117, CD56, CD10, PDGFRβ, PDPN, CD90, FAP, and CD29. tSNE analysis is depicted, and the subset noted as missing from culture is denoted with a box gate. B. CD73 staining of human FRCs; assessed culture passage 0, 1, or 4; or freshly isolated and gated as CD45^−^ CD31^−^ EpCAM^−^ PDPN^+^, C. Human tonsil–derived FRCs, at various passages or freshly isolated, were gated as CD45^−^ CD31^−^ EpCAM^−^ and assessed for expression of CD90 and PDPN. D. Expression of FRC-relevant genes from RNA-seq, represented as a heatmap. Colour gradation denotes the relative gene expression level of selected genes normalised from 0 to 1, while the size of the circles denotes TPM. The absence of a circle denotes no detectable transcripts, seen for CR2, CCL21, CCL19, CXCL9, and CXCL10. Note that relatively low transcription of PDPN mRNA nonetheless yields strong expression of the glycoprotein, as shown in C. αSMA, α smooth muscle actin; CCL19, chemokine C-C motif ligand 19; CCL21, chemokine C-C motif ligand 21; CR2, complement receptor type 2; CXCL9, chemokine C-X-C motif ligand 9; CXCL10, chemokine C-X-C motif ligand 10; FAP, fibroblast activation protein; FRC, fibroblastic reticular cell; PDGFRβ, platelet-derived growth factor receptor beta; PDPN, podoplanin; RNA-seq, RNA sequencing; TPM, transcripts per million; tSNE, t-distributed stochastic neighbour embedding.
(TIF)

S2 Fig The effect of FRCs on T cells in G0/G1 and G2/M phase of cell cycle. CFSE-labelled PBMCs (5 × 10^5^) were stimulated with anti-CD3/CD28/CD2-coated beads, with or without inhibitors. After 96 h, cells were harvested and analysed by flow cytometry. Flow cytometric cell cycle analysis of A. CD4 T cells and B. CD8 T cells was performed using BrdU and 7AAD to assess percentage of cells in G0/G1 phase and G2/M phase. Figure is representative of *N* = 4 FRC donors and *N* = 2 PBMC donors from 2 independent experiments. Box and whisker plots are shown. Data used in the generation of this figure can be found in S1 Data. 7AAD, 7-aminoactinomycin D; BrdU, bromodeoxyuridine; CFSE, carboxyfluorescein succinimidyl ester; FRC, fibroblastic reticular cell; PBMC, peripheral blood mononuclear cell.
(TIF)

S3 Fig The effect of inhibitors on T-cell stimulation. CFSE-labelled PBMCs (5 × 10^5^) were stimulated with anti-CD3/CD28/CD2-coated beads, with or without inhibitors. After 96 h, cells were harvested and analysed by flow cytometry. Plots were gated for CD3, CD4, or CD8; CD62L; and CD45RO. A. Fold change in the proportion of CD4^+^ T cells that are naïve (CD62L^+^CD45RO^−^), effector (‘Eff’, CD62L^−^CD45RO^−^), central memory (‘CM’, CD62L^+^CD45RO^+^), or effector memory (‘EM’, CD62L^−^CD45RO^+^), comparing stimulated (‘Stim’) T cells + inhibitors to stimulated T cells without inhibitors. B. Fold change in the proportion of CD8^+^ T cells that are naïve, effector, central memory, or effector memory, comparing stimulated (‘Stim’) T cells + inhibitors to stimulated T cells without inhibitors. Figure depicts 6–7 FRC donors and 6 PBMC donors from 6 independent experiments. Data used in the generation of this figure can be found in S1 Data. CFSE, carboxyfluorescein succinimidyl ester; FRC, fibroblastic reticular cell; PBMC, peripheral blood mononuclear cell.
(TIF)

S4 Fig All 4 suppressive mechanisms are utilised in all donors. FRCs were cocultured with PBMCs stimulated using anti-CD3/CD28/CD2-coated beads, with or without individual inhibitors for 96 h prior to harvest and analysis. *N* = 4 FRC donors and *N* = 1 PBMC donor. The *y* axis depicts the division index for gated CD8 T cells with or without FRCs and with or without inhibitors, normalised to the value of stimulated T cells in the presence of FRCs (maximal suppression = 1). Data used in the generation of this figure can be found in S1 Data. FRC, fibroblastic reticular cell; PBMC, peripheral blood mononuclear cell.
(TIF)

S5 Fig Further profiling and relative proliferation of memory phenotype cells. A. CFSE-labelled PBMCs were incubated with or without anti-CD3/CD28/CD2-coated beads for 96 h prior to harvest and analysis. Central memory (gated as CD3+CD62L+CD45RO+) and effector memory cells (gated as CD3+CD62L-CD45RO+) were identified, and the relative proliferative capacity of central versus effector memory T cells was examined through CFSE dilution. B. CD27 staining (blue dots) was projected onto a plot gated on CD3+ single lymphocytes, showing specificity for central memory and naïve T cells, while effector memory and effector T cells were CD27 negative. Data represent *n* = 2 PBMC donors. CFSE, carboxyfluorescein succinimidyl ester; PBMC, peripheral blood mononuclear cell.
(TIF)

S6 Fig T cells activated in the presence of pre-inhibited FRCs do not show reduced CD25 expression. FRCs were pre-incubated with inhibitors for 4 h and then washed thoroughly in PBS prior to coculture with T cells and activating anti-CD3/CD28/CD2-coated beads. Activation proceeded for 24 h before T cells were harvested for flow cytometric analysis. CD25 expression was examined as a proxy for activation. Figure is representative of *N* = 2 FRC donors and 1 PBMC donor. Data used in the generation of this figure can be found in S1 Data. FRC, fibroblastic reticular cell; PBMC, peripheral blood mononuclear cell.
(TIF)

S7 Fig PhosFlow experimental analysis of transcription factors of T cells. PhosFlow experimental analysis of transcription factors of T cells, gated using CD3 and A. CD4 or B. CD8. T cells were activated using anti-CD3/CD28/CD2-coated beads and cocultured with FRCs and/or inhibitor cocktail. Figure depicts results from 1 experiment using 1 PBMC donor and *N* = 2 FRC donors and is representative of 2 independent experiments utilising *N* = 4 FRC donors and *N* = 2 PBMC donors. Data used in the generation of this figure can be found in S1 Data. FRC, fibroblastic reticular cell; PBMC, peripheral blood mononuclear cell.
(TIF)

S8 Fig Tregs are not required for FRC-mediated suppression. A. PBMCs were incubated with or without FRCs and with or without anti-CD3/CD28/CD2-coated beads for 96 h prior to harvest and analysis. Gating strategy for CD3+4+127lo/neg CD25+FoxP3+ Tregs is shown. B. From the experiment described in A, aggregate data from 3 independent experiments, 3 FRC donors, and 3 PBMC donors are shown. Depicted are Tregs (CD3+CD4+CD25+CD127lo/neg FoxP3+ cells) measured as a percentage of live lymphocytes; Tregs measured as a percentage of CD4 T cells; CD127lo/negCD25+ cells as a percentage of CD4+T cells; Tregs as a percentage of CD127lo/negCD25+ cells and the overall ratio of CD4+ T cells to Tregs. Lines connect individual experiments. Data depict 3 independent experiments, 3 FRC donors, and 3 PBMC donors. C. PBMCs were either magnetically depleted of CD25+ cells or left undepleted, prior to activation using anti-CD3/CD28/CD2-coated beads and culture with FRCs to remove Tregs. Depletion of CD25+ cells did not prevent FRCs from suppressing T cells at 96 h. Plots gated as CD3+ and either CD4+ or CD8+ as shown. Data represent 2 FRC donors and 2 PBMC donors from 2 individual experiments. Data used in the generation of this figure can be found in S1 Data. FoxP3, forkhead box P3; FRC, fibroblastic reticular cell; PBMC, peripheral blood mononuclear cell; Treg, regulatory T cell.
(TIF)

S9 Fig COX2, A2AR, and TGFβR2 protein staining in TRCs. Tonsil sections were stained for CD3 and for the antigen identified by antibody clone ERTR7 and A2AR, COX2, TGFBR2, or a PBS/serum + secondary antibody control. Data represent 3–5 sections per donor from 3 donors. Scale bars represent 50 μm. Arrows denote areas of colocalisation between ERTR7 and either A2AR, COX2, or TGFBR2. A2AR, adenosine 2A receptor; COX2, cyclooxygenase-2; TGFβR2, transforming growth factor beta receptor type 2; TRC, T-zone fibroblastic reticular cell.
(TIF)

## Abbreviations

1-MT: 1-methyl-D-Tryptophan
7AAD: 7-aminoactinomycin D
αSMA: α smooth muscle actin
A2AR: adenosine 2A receptor
ALK5: activin receptor-like kinase 5
ATP: adenosine triphosphate
BAFF: B cell activating factor
BrdU: bromodeoxyuridine
BSA: bovine serum albumin
CAR: chimeric antigen receptor
CCL19: chemokine C-C motif ligand 19
CCL21: chemokine C-C motif ligand 21
CFSE: carboxyfluorescein succinimidyl ester
CHO: Chinese hamster ovary
COX2: cyclooxygenase-2
CXCL13: chemokine C-X-C motif ligand 13
FRC: fibroblastic reticular cell
HBRC: Human Biomaterials Resource Centre
hCLEC: human C-type lectin domain family 14 member A
IDO: indoleamine-2,3-dioxygenase
IFNγ: interferon gamma
IL-2Ra: interleukin 2 receptor alpha
IL-6: interleukin 6
IL-7: interleukin 7
LTBR: lymphotoxin beta receptor
MADCAM1: mucosal vascular addressin cell adhesion molecule 1
MAPK: mitogen-activated protein kinase
mTOR: mammalian target of rapamycin
NDRI: National Disease Research Interchange
NK: natural killer
NOS2: nitric oxide synthase 2
PBMC: peripheral blood mononuclear cell
PD-1: programmed cell death protein 1
PDGFR: platelet-derived growth factor receptor
PD-L1: programmed cell death ligand 1
PDPN: podoplanin
PECAM1: platelet and endothelial cell adhesion molecule 1
pERK1/2: phosphorylated extracellular signal–regulated kinase 1/2
PGE2: prostaglandin E2
PHA-L: phytohemagglutinin-L
pSTAT1: phosphorylated signal transducer and activator of transcription 1
pSTAT3: phosphorylated signal transducer and activator of transcription 3
pSTAT5: phosphorylated STAT5
pSTAT6: phosphorylated signal transducer and activator of transcription 6
PTPRC: protein tyrosine phosphatase receptor type C
pTreg: peripheral Treg
PTSG1: prostaglandin-endoperoxide synthase 1
PTSG2: prostaglandin-endoperoxide synthase 2
RANKL: receptor activator of NF-κB ligand
rhIL-2: recombinant human interleukin 2
RT: room temperature
S phase: DNA-synthesis phase
STAT5: signal transducer and activator of transcription 5
TGFβRI: transforming growth factor beta receptor type 1
TGFβR2: transforming growth factor beta receptor type 2
Th1: T helper 1
Th2: T helper 2
Th17: T helper 17
TMB: 3,3′,5,5′-tetramethylbenzidine
TNFα: tumour necrosis factor alpha
TRC: T-zone FRC
Treg: T regulatory cell
VIM: vimentin
WT: wild type
