# Supplementary figures and images for: The human lymph node microenvironment unilaterally regulates T-cell activation and differentiation
Source: PLoS Biol. 2018 Sep 4;16(9):e2005046. doi: 10.1371/journal.pbio.2005046 (PMC6122729; doi:10.1371/journal.pbio.2005046)

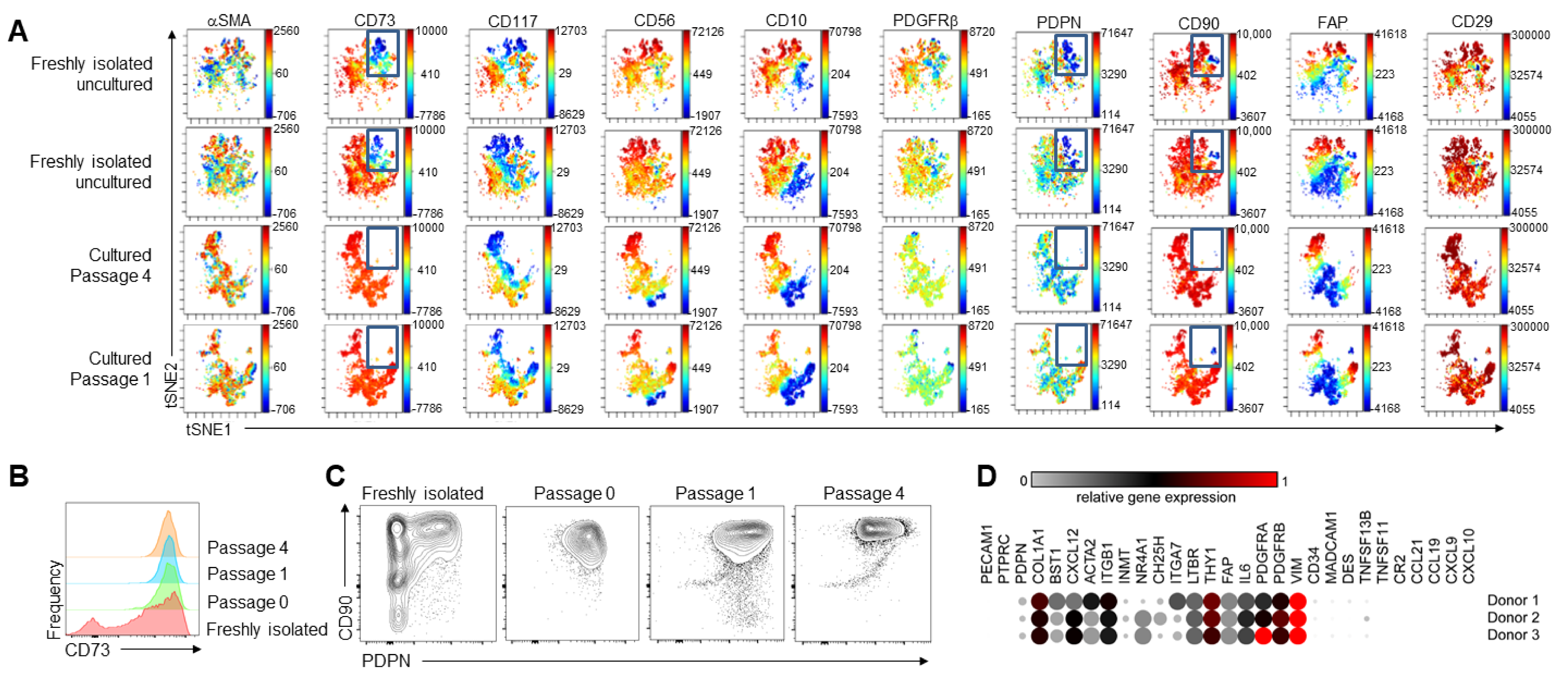

Supplement: S1 Fig — A. Human tonsil–derived FRCs, at various passages or freshly isolated, were gated as CD45− CD31− EpCAM− and assessed by flow cytometry for expression of αSMA, CD73, CD117, CD56, CD10, PDGFRβ, PDPN, CD90, FAP, and CD29. tSNE analysis is depicted, and the subset noted as missing from culture is denoted with a box gate. B. CD73 staining of human FRCs; assessed culture passage 0, 1, or 4; or freshly isolated and gated as CD45− CD31− EpCAM− PDPN+, C. Human tonsil–derived FRCs, at various passages or freshly isolated, were gated as CD45− CD31− EpCAM− and assessed for expression of CD90 and PDPN. D. Expression of FRC-relevant genes from RNA-seq, represented as a heatmap. Colour gradation denotes the relative gene expression level of selected genes normalised from 0 to 1, while the size of the circles denotes TPM. The absence of a circle denotes no detectable transcripts, seen for CR2, CCL21, CCL19, CXCL9, and CXCL10. Note that relatively low transcription of PDPN mRNA nonetheless yields strong expression of the glycoprotein, as shown in C. αSMA, α smooth muscle actin; CCL19, chemokine C-C motif ligand 19; CCL21, chemokine C-C motif ligand 21; CR2, complement receptor type 2; CXCL9, chemokine C-X-C motif ligand 9; CXCL10, chemokine C-X-C motif ligand 10; FAP, fibroblast activation protein; FRC, fibroblastic reticular cell; PDGFRβ, platelet-derived growth factor receptor beta; PDPN, podoplanin; RNA-seq, RNA sequencing; TPM, transcripts per million; tSNE, t-distributed stochastic neighbour embedding. (TIF) [file pbio.2005046.s003.tif]

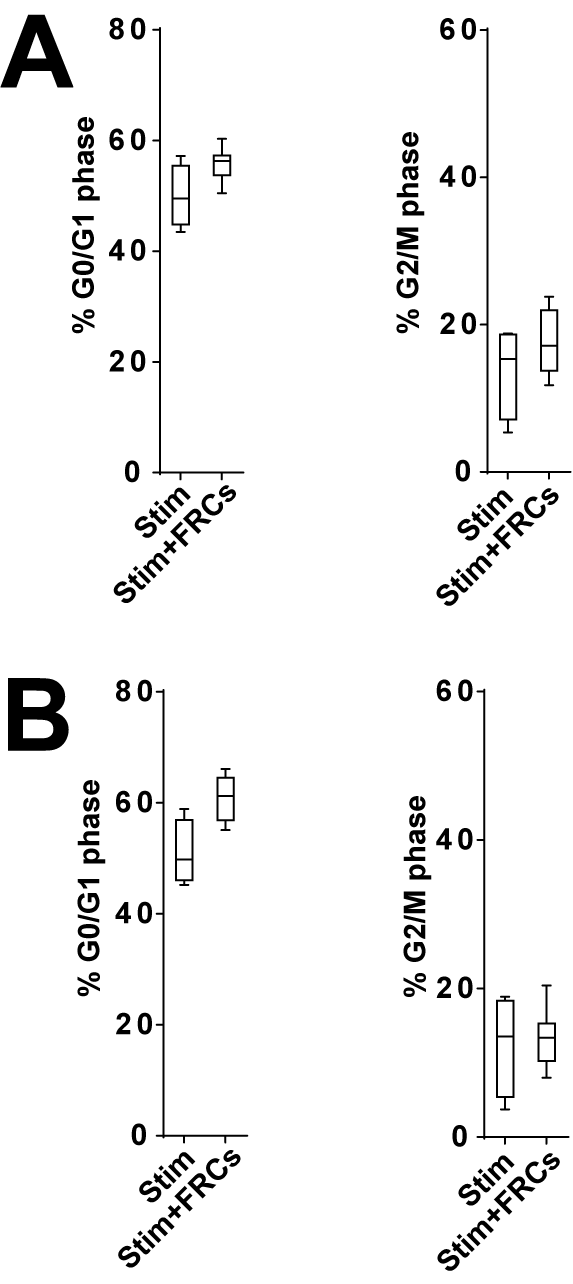

Supplement: S2 Fig — CFSE-labelled PBMCs (5 × 10^5^) were stimulated with anti-CD3/CD28/CD2-coated beads, with or without inhibitors. After 96 h, cells were harvested and analysed by flow cytometry. Flow cytometric cell cycle analysis of A. CD4 T cells and B. CD8 T cells was performed using BrdU and 7AAD to assess percentage of cells in G0/G1 phase and G2/M phase. Figure is representative of N = 4 FRC donors and N = 2 PBMC donors from 2 independent experiments. Box and whisker plots are shown. Data used in the generation of this figure can be found in S1 Data. 7AAD, 7-aminoactinomycin D; BrdU, bromodeoxyuridine; CFSE, carboxyfluorescein succinimidyl ester; FRC, fibroblastic reticular cell; PBMC, peripheral blood mononuclear cell. (TIF) [file pbio.2005046.s004.tif]

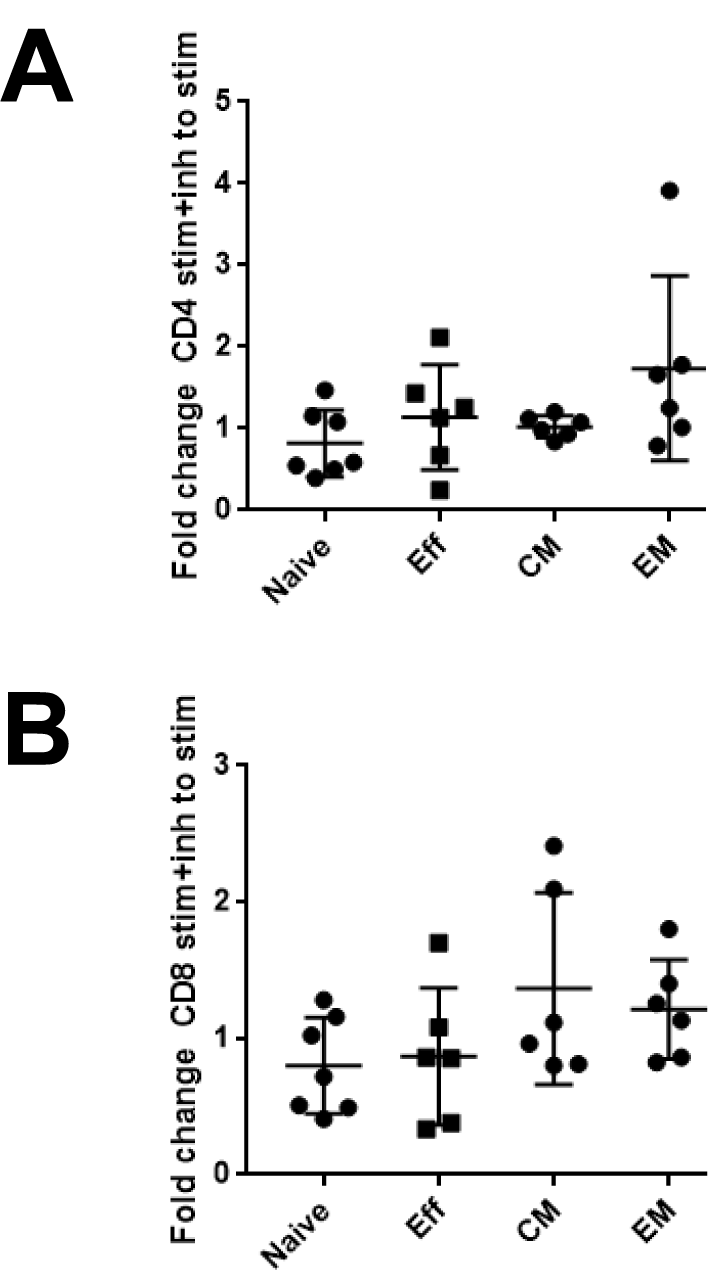

Supplement: S3 Fig — CFSE-labelled PBMCs (5 × 10^5^) were stimulated with anti-CD3/CD28/CD2-coated beads, with or without inhibitors. After 96 h, cells were harvested and analysed by flow cytometry. Plots were gated for CD3, CD4, or CD8; CD62L; and CD45RO. A. Fold change in the proportion of CD4+ T cells that are naïve (CD62L+CD45RO−), effector (‘Eff’, CD62L−CD45RO−), central memory (‘CM’, CD62L+CD45RO+), or effector memory (‘EM’, CD62L−CD45RO+), comparing stimulated (‘Stim’) T cells + inhibitors to stimulated T cells without inhibitors. B. Fold change in the proportion of CD8+ T cells that are naïve, effector, central memory, or effector memory, comparing stimulated (‘Stim’) T cells + inhibitors to stimulated T cells without inhibitors. Figure depicts 6–7 FRC donors and 6 PBMC donors from 6 independent experiments. Data used in the generation of this figure can be found in S1 Data. CFSE, carboxyfluorescein succinimidyl ester; FRC, fibroblastic reticular cell; PBMC, peripheral blood mononuclear cell. (TIF) [file pbio.2005046.s005.tif]

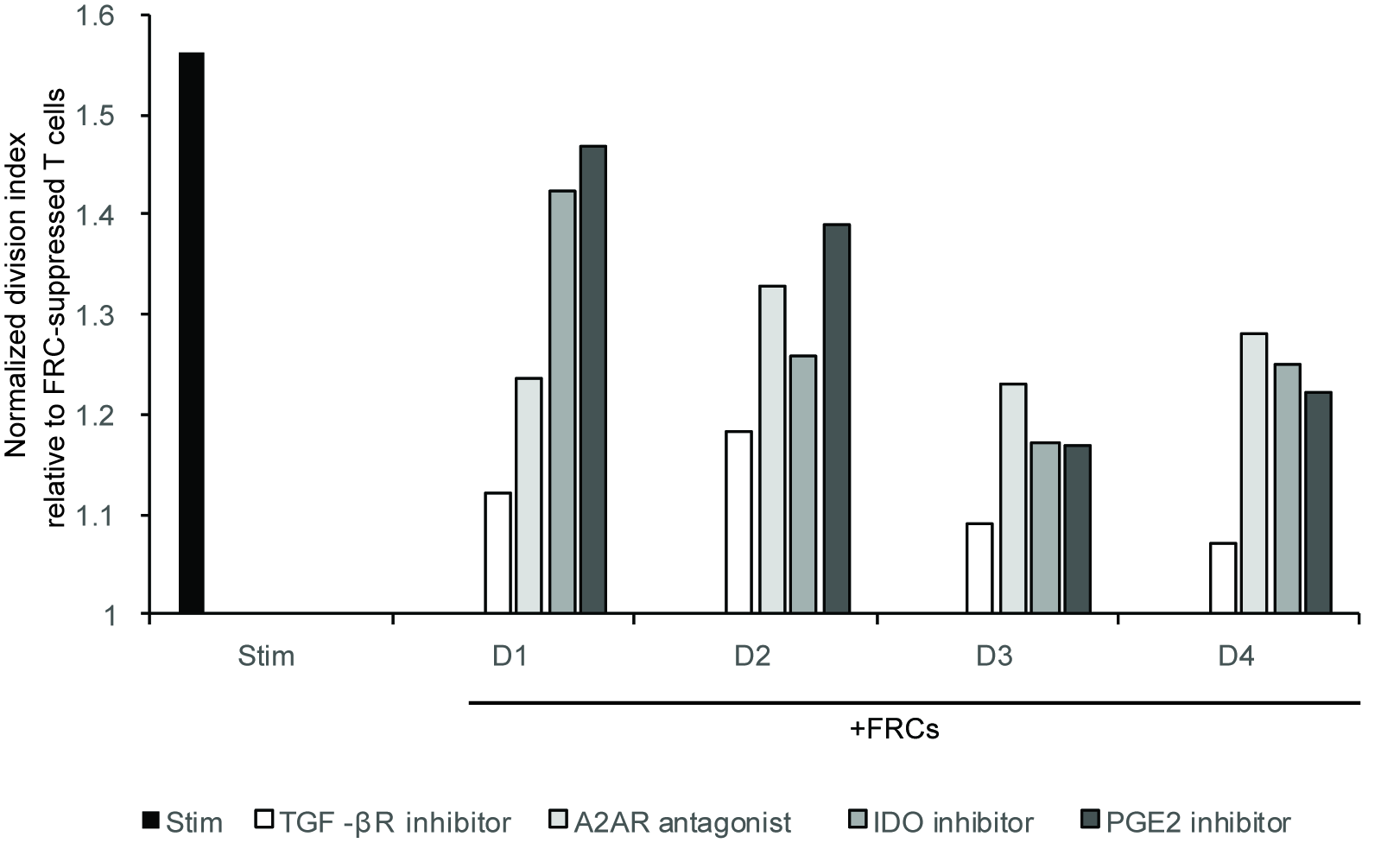

Supplement: S4 Fig — FRCs were cocultured with PBMCs stimulated using anti-CD3/CD28/CD2-coated beads, with or without individual inhibitors for 96 h prior to harvest and analysis. N = 4 FRC donors and N = 1 PBMC donor. The y axis depicts the division index for gated CD8 T cells with or without FRCs and with or without inhibitors, normalised to the value of stimulated T cells in the presence of FRCs (maximal suppression = 1). Data used in the generation of this figure can be found in S1 Data. FRC, fibroblastic reticular cell; PBMC, peripheral blood mononuclear cell. (TIF) [file pbio.2005046.s006.tif]

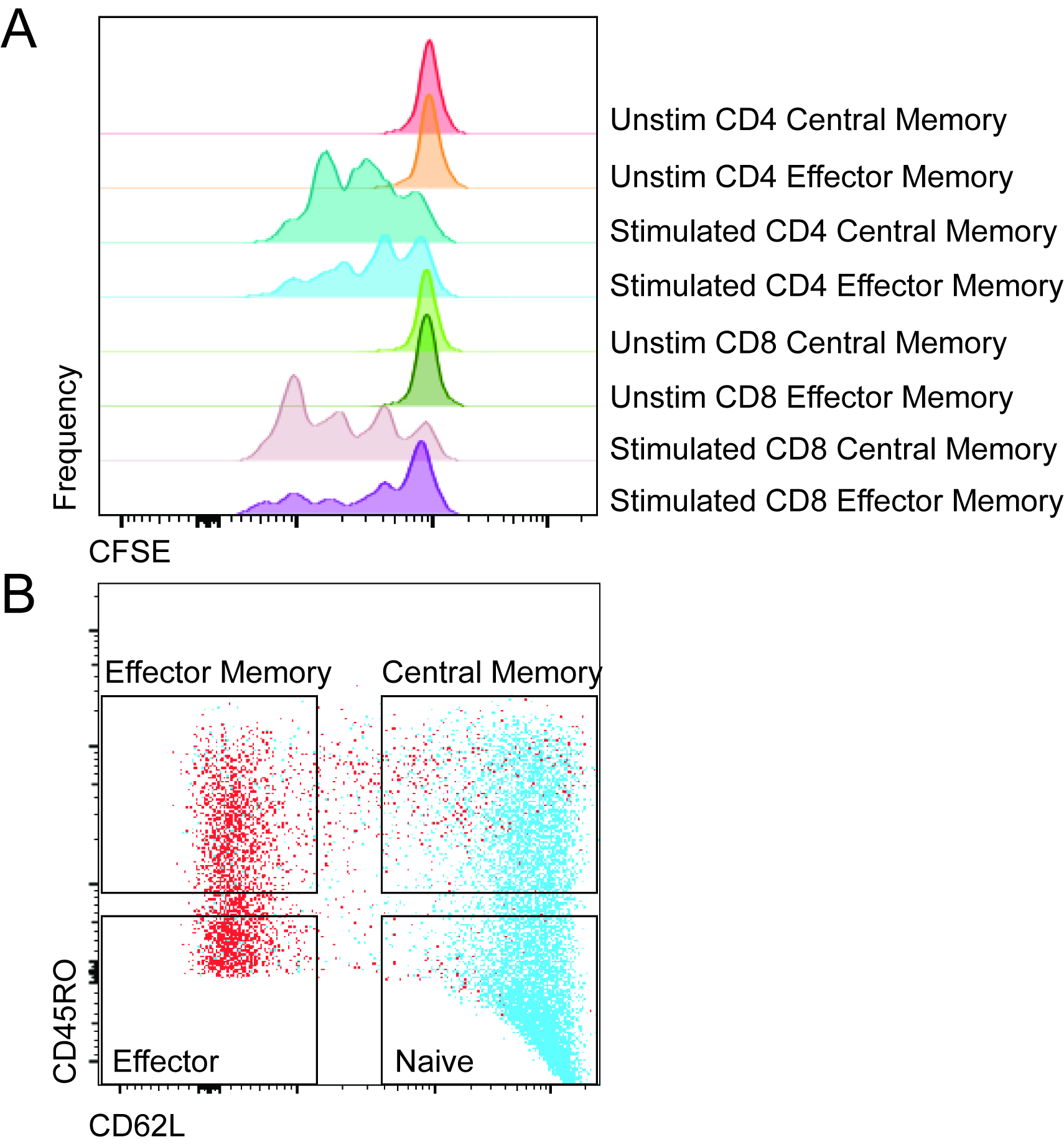

Supplement: S5 Fig — A. CFSE-labelled PBMCs were incubated with or without anti-CD3/CD28/CD2-coated beads for 96 h prior to harvest and analysis. Central memory (gated as CD3+CD62L+CD45RO+) and effector memory cells (gated as CD3+CD62L-CD45RO+) were identified, and the relative proliferative capacity of central versus effector memory T cells was examined through CFSE dilution. B. CD27 staining (blue dots) was projected onto a plot gated on CD3+ single lymphocytes, showing specificity for central memory and naïve T cells, while effector memory and effector T cells were CD27 negative. Data represent n = 2 PBMC donors. CFSE, carboxyfluorescein succinimidyl ester; PBMC, peripheral blood mononuclear cell. (TIF) [file pbio.2005046.s007.tif]

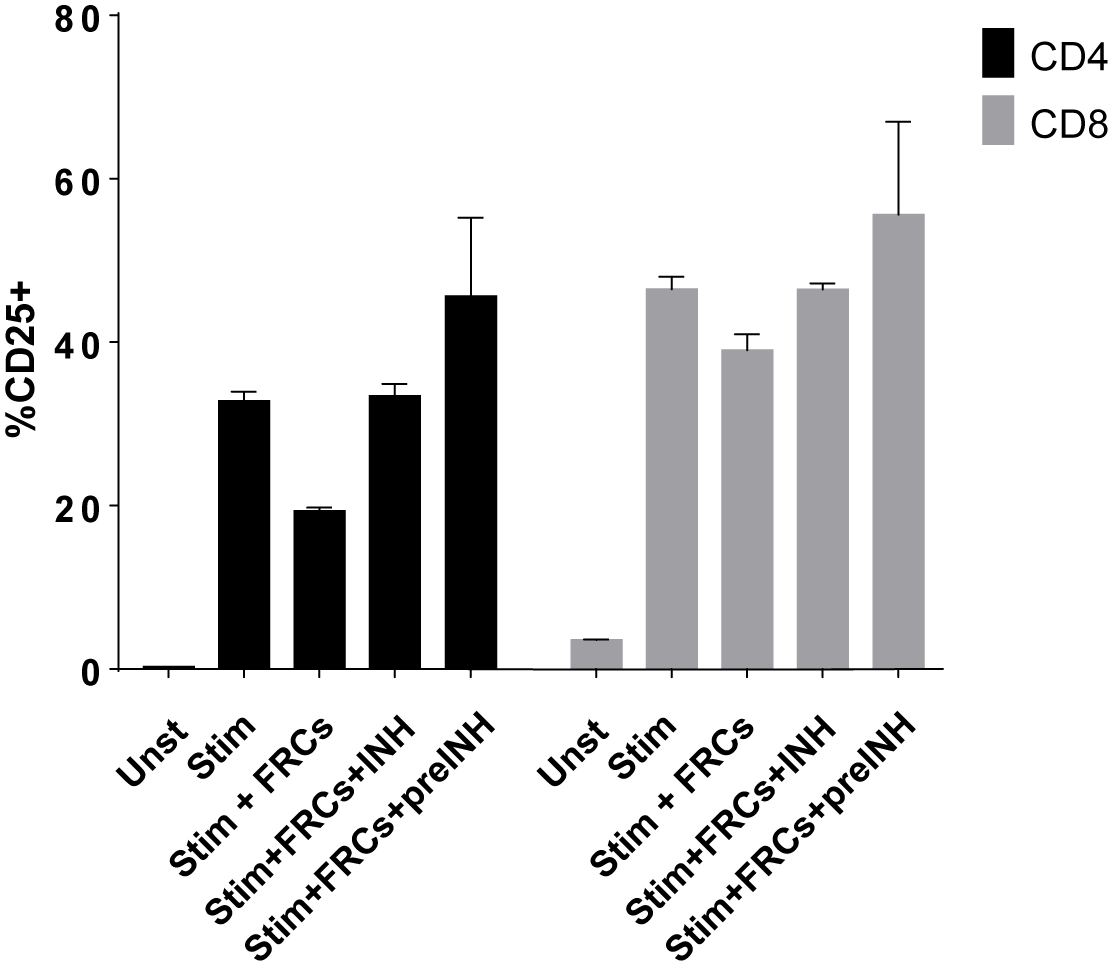

Supplement: S6 Fig — FRCs were pre-incubated with inhibitors for 4 h and then washed thoroughly in PBS prior to coculture with T cells and activating anti-CD3/CD28/CD2-coated beads. Activation proceeded for 24 h before T cells were harvested for flow cytometric analysis. CD25 expression was examined as a proxy for activation. Figure is representative of N = 2 FRC donors and 1 PBMC donor. Data used in the generation of this figure can be found in S1 Data. FRC, fibroblastic reticular cell; PBMC, peripheral blood mononuclear cell. (TIF) [file pbio.2005046.s008.tif]

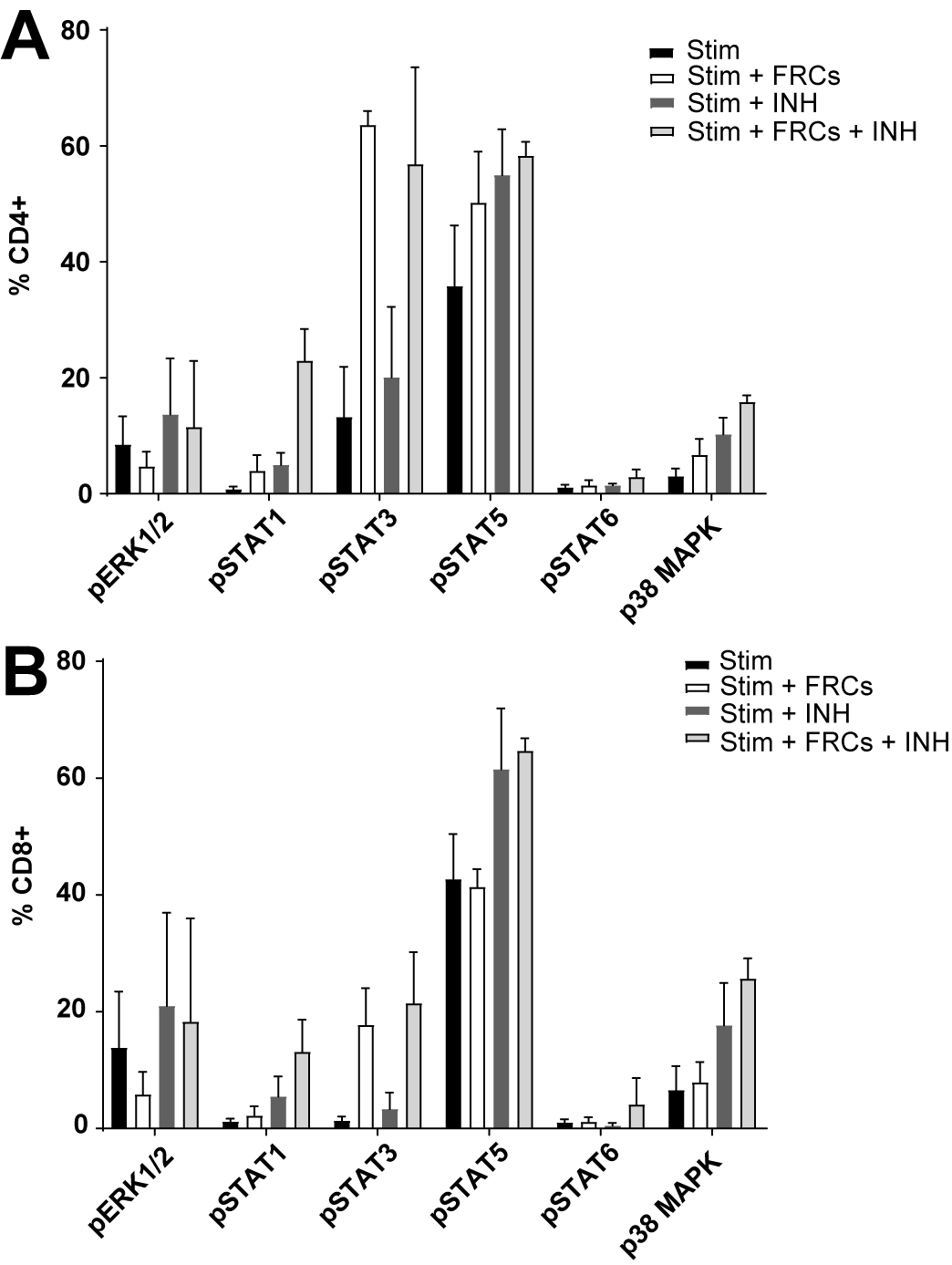

Supplement: S7 Fig — PhosFlow experimental analysis of transcription factors of T cells, gated using CD3 and A. CD4 or B. CD8. T cells were activated using anti-CD3/CD28/CD2-coated beads and cocultured with FRCs and/or inhibitor cocktail. Figure depicts results from 1 experiment using 1 PBMC donor and N = 2 FRC donors and is representative of 2 independent experiments utilising N = 4 FRC donors and N = 2 PBMC donors. Data used in the generation of this figure can be found in S1 Data. FRC, fibroblastic reticular cell; PBMC, peripheral blood mononuclear cell. (TIF) [file pbio.2005046.s009.tif]

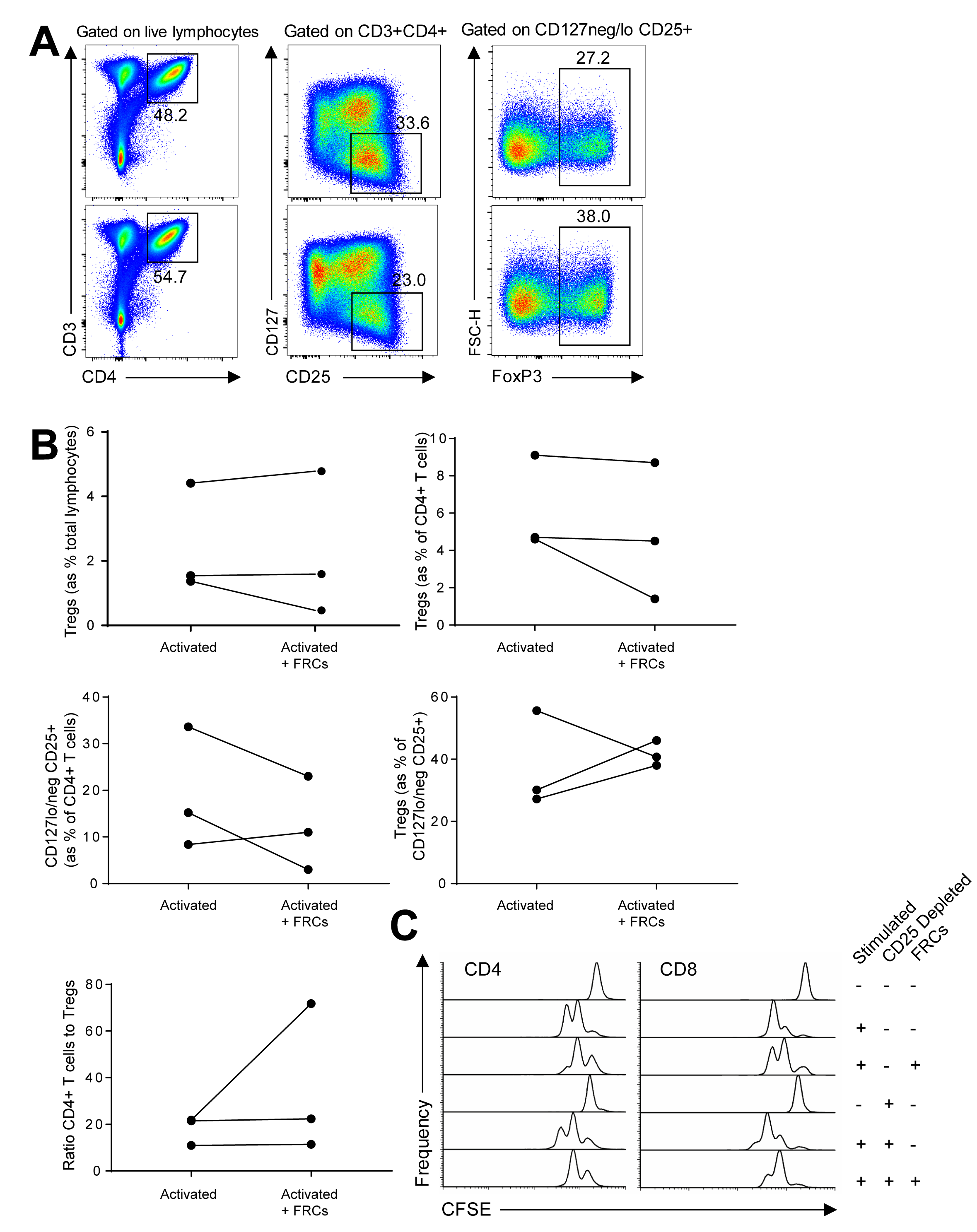

Supplement: S8 Fig — A. PBMCs were incubated with or without FRCs and with or without anti-CD3/CD28/CD2-coated beads for 96 h prior to harvest and analysis. Gating strategy for CD3+4+127lo/neg CD25+FoxP3+ Tregs is shown. B. From the experiment described in A, aggregate data from 3 independent experiments, 3 FRC donors, and 3 PBMC donors are shown. Depicted are Tregs (CD3+CD4+CD25+CD127lo/neg FoxP3+ cells) measured as a percentage of live lymphocytes; Tregs measured as a percentage of CD4 T cells; CD127lo/negCD25+ cells as a percentage of CD4+T cells; Tregs as a percentage of CD127lo/negCD25+ cells and the overall ratio of CD4+ T cells to Tregs. Lines connect individual experiments. Data depict 3 independent experiments, 3 FRC donors, and 3 PBMC donors. C. PBMCs were either magnetically depleted of CD25+ cells or left undepleted, prior to activation using anti-CD3/CD28/CD2-coated beads and culture with FRCs to remove Tregs. Depletion of CD25+ cells did not prevent FRCs from suppressing T cells at 96 h. Plots gated as CD3+ and either CD4+ or CD8+ as shown. Data represent 2 FRC donors and 2 PBMC donors from 2 individual experiments. Data used in the generation of this figure can be found in S1 Data. FoxP3, forkhead box P3; FRC, fibroblastic reticular cell; PBMC, peripheral blood mononuclear cell; Treg, regulatory T cell. (TIF) [file pbio.2005046.s010.tif]

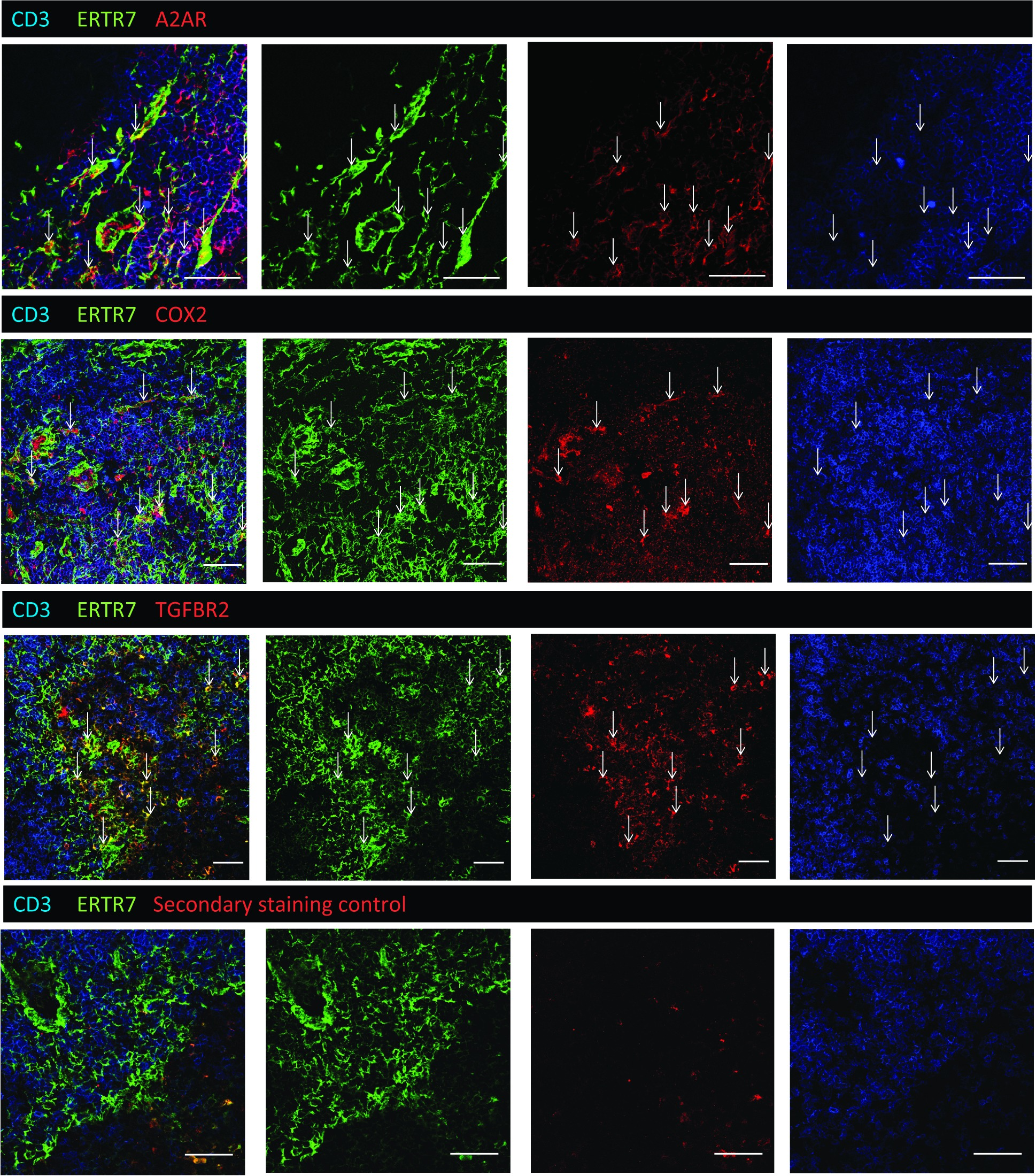

Supplement: S9 Fig — Tonsil sections were stained for CD3 and for the antigen identified by antibody clone ERTR7 and A2AR, COX2, TGFBR2, or a PBS/serum + secondary antibody control. Data represent 3–5 sections per donor from 3 donors. Scale bars represent 50 μm. Arrows denote areas of colocalisation between ERTR7 and either A2AR, COX2, or TGFBR2. A2AR, adenosine 2A receptor; COX2, cyclooxygenase-2; TGFβR2, transforming growth factor beta receptor type 2; TRC, T-zone fibroblastic reticular cell. (TIF) [file pbio.2005046.s011.tif]
